# Supplementary material for: Regional sex differences in human cortical anatomy vary in their morphometric bases and overlap with sex chromosomal and gonadal influences
Source: Nat Commun. 2026 Jun 13;17:7503. doi: 10.1038/s41467-026-74274-8 (PMC13408908; doi:10.1038/s41467-026-74274-8)
Supplement: Supplementary file 1 — Supplementary Information [file 41467_2026_74274_MOESM1_ESM.pdf]

Supplementary Information for "Regional sex differences in human cortical anatomy vary in their morphometric bases and overlap with sex chromosomal and gonadal influences"

Hyo M. Lee, PhD[1]; Siyuan Liu, PhD[1]; Elisa Guma, PhD[1,2]; Elizabeth Levitis, PhD[1,3,4]; Rebecca Shafee, PhD[1]; Gabrielle Dugan, BS[1]; François M. Lalonde, PhD[1]; Liv Clasen, PhD[1]; Alex DeCasien, PhD[1,5]; M. Mallar Chakravarty, PhD[6,7]; Jason P. Lerch, PhD[8,9,10,11]; Konrad Wagstyl, MD, PhD[12,13]; Angela Delaney, MD[14,15]; Armin Raznahan, MD, PhD[1]

1. Section on Developmental Neurogenomics, National Institute of Mental Health, Bethesda, Maryland, United States

2. Department of Pediatrics, Massachusetts General Hospital, Boston, Massachusetts, United States

3. Center for Medical Image Computing, Department of Computer Science, University College London, London, United Kingdom

4. Department of Child and Adolescent Psychiatry and Behavioral Science, The Children's Hospital of Philadelphia, Philadelphia, PA, USA

5. Laboratory of Neurogenetics, National Institute on Aging, Bethesda, Maryland, United States

6. Department of Psychiatry, McGill University, Montreal, Quebec, Canada

7. Cerebral Imaging Centre, Douglas Research Centre, Montreal, Quebec, Canada

8. Mouse Imaging Centre, Toronto, Canada

9. The Hospital for Sick Children, Toronto, Canada

10. Department of Medical Biophysics, University of Toronto, Toronto, Canada

11. Wellcome Centre for Integrative Neuroimaging, Nuffield Department of Clinical Neurosciences, University of Oxford, Oxford, United Kingdom

12. School of Biomedical Engineering & Imaging Sciences, King's College London, London, United Kingdom

13. UCL Great Ormond Street Institute of Child Health, London, United Kingdom

14. Division of Endocrinology, Department of Pediatric Medicine, St. Jude Children's Research Hospital, Memphis, Tennessee, United States

15. Department of Epidemiology and Cancer Control, St. Jude Children's Research Hospital, Memphis, Tennessee, United States

	HCP		UKB		SCA XXY		SCA XYY		IGD	
	**Female**	**Male**	**Female**	**Male**	**XXY**	**XY**	**XYY**	**XY**	**IGD XY**	**XY**
Sample size (n)	592	493	375	294	99	92	34	47	19	22
Age (mean ± SD)	29.56 ± 3.6	27.89 ± 3.59	48.75 ± 0.94	48.89 ± 0.83	16.38 ± 4.78	16.24 ± 5.64	15.49 ± 5.33	13.97 ± 4.76	23.27 ± 8.33	21.89 ± 7.66

Supplementary Table 1. Demographic characteristics of Human Connectome Project (HCP), UK Biobank (UKB), Sex Chromosome Aneuploidy (SCA) and isolated gonadotropin-releasing hormone (GnRH) deficiency (IGD) datasets.

Analysis	Statistical Tests Applied
Figures 1B, 2C	Comparison of cortical maps using both one-tailed Spin and participant permutation tests (P_{SPIN} and P_{PERM}, respectively), with subsequent Bonferroni correction of these empirical p values ($P_{SPIN-BF}$ and $P_{PERM-BF} < 0.05$) across the 3 cortical features: CV, SA and CT.
Figures 1C, 2D, Figure 4A, 4B, 4C (first rows), Sup Figure 5A, 5B, 5C (first rows)	The statistical significance of group (females vs. males, XXY vs. XY, XYY vs. XY, typical XY vs. XY IGD) effects based on a two-tailed t-test is controlled for multiple comparisons using FDR correction ($p_{FDR}<0.05$) across 360 cortical regions.
Figures 2E, Figure 4A, 4B, 4C (second rows), Sup Figure 5A, 5B, 5C (second rows)	Proportion of cortical sheets in agreement or congruence using one-tailed Spin permutation test (p_{SPIN}), with subsequent Bonferroni correction of these empirical p values ($P_{SPIN-BF}<0.05$) across the 3 cortical features, CV, SA and CT.
Figure 3A, Sup Figure 4A	The mean standardized effect sizes of cortical sex differences within each of the 17 functional networks using two-tailed Spin permutation test (p_{SPIN}), with subsequent Bonferroni correction ($p_{SPIN-BF}<0.05$) across 3 test cases (CV, SA and CT).
Figure 3B, Sup Figure 4B	Gene set enrichment using one-tailed Spin permutation test (p_{SPIN}), with subsequent Bonferroni correction ($p_{SPIN-BF}<0.05$) across 6 test cases (female-bases vs. male-biases for CV, SA and CT).
Sup Figure 1	Comparison of global phenotypes using linear correlation (two-tailed p), with subsequent Bonferroni correction across the 3 cortical features: CV, SA and CT.

Supplementary Table 2. Summary of statistical testing and multiple correction methods used in this study.

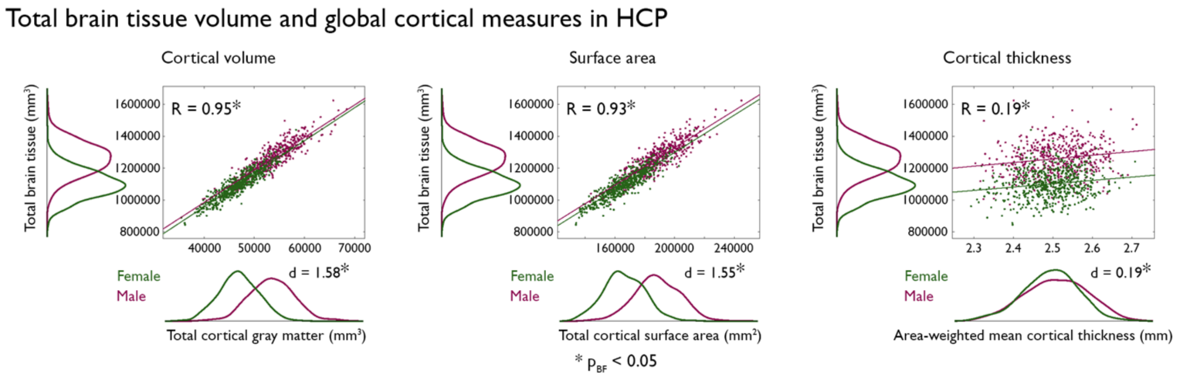

Supplementary Figure 1. Relationships between total brain tissue volume and global surface-based measures of cortical anatomy show the sex difference in each global phenotype and the limitations of total tissue volume as a global size covariate in analysis of cortical thickness. Scatter plots showing the relationship between interindividual variation in total brain tissue volume and global measures of cortical volume, surface area and thickness (females – green; males – pink), with least squares linear regression fit lines in each sex. Marginal histograms show the distribution of each feature in each sex, with Cohen's effect size (d) comparing each feature between the sexes, and asterisks denoting Bonferroni-corrected two-tailed statistical significance across plots at 0.05. The Pearson correlation (R) between features is shown for each plot with asterisks denoting Bonferroni-corrected statistical significance across plots at 0.05. The low R value for cortical thickness shows why total brain volume is not an equally effective covariate for control of global size in analyses of cortical volume, surface area and thickness.

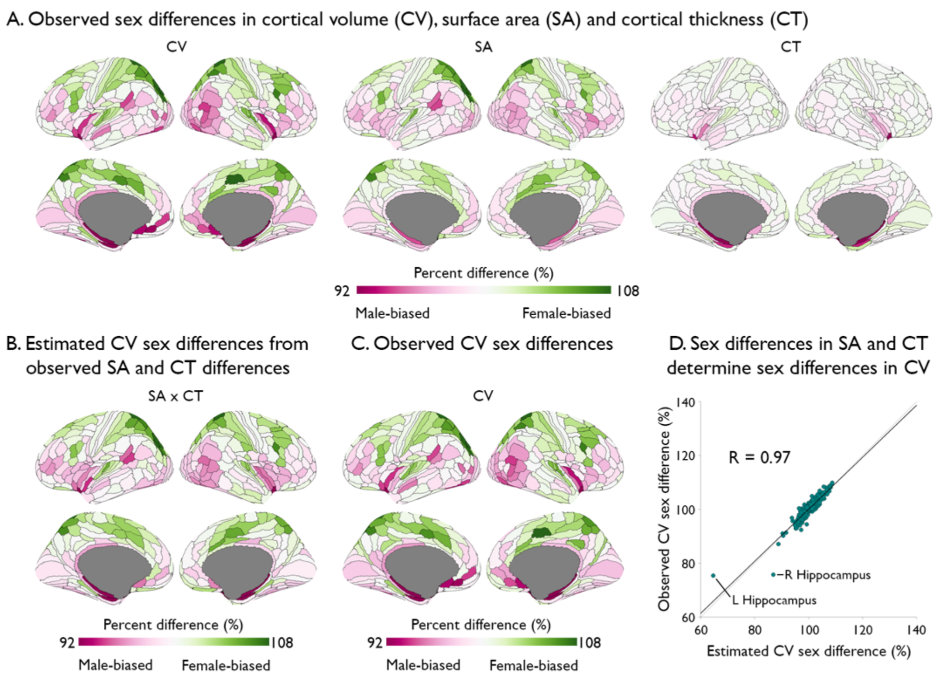

Supplementary Figure 2. Sex differences in cortical volume (CV) are the product of sex differences in cortical surface area and thickness (SA and CT). **A**. Observed percent sex difference in CV, SA and CT across 360 cortical regions (female-biases in green, male-biases in pink). **B.** The product of percentage difference maps for SA and CT. **C.** Observed percent sex difference in CV across 360 cortical regions from A. **D.** A scatter plot verifying the expected high concordance (Pearson R = 0.97) between the observed percentage sex difference in regional CV (y-axis) and the multiplication of percentage sex difference maps for SA and CT (x-axis). The solid black line is the least squares linear regression fit line – which perfectly overlays and therefore obscures the dotted line x=y identity line. Note that directly estimated sex differences in CV are least well predicted by observed sex differences in SA and CT for the left and right hippocampus.

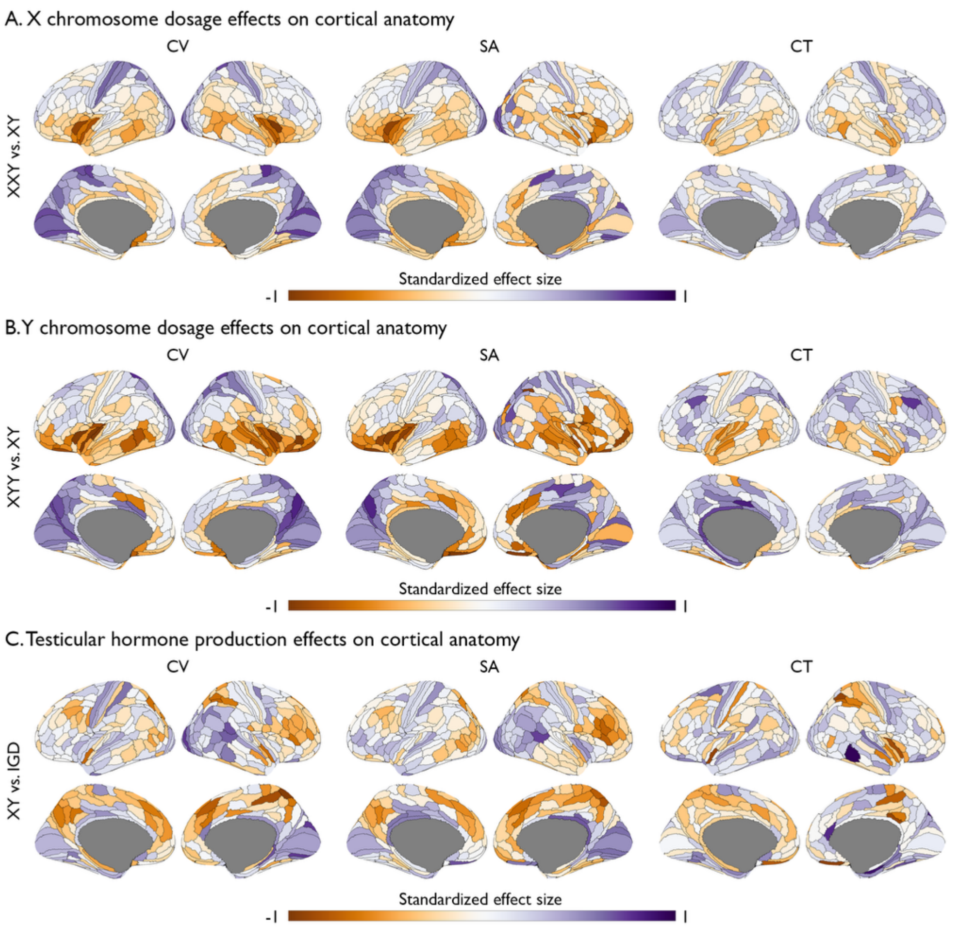

Supplementary Figure 3. The effects of X and Y chromosome dosage and testicular hormone production on regional cortical volume (CV), area (SA) and thickness (CT). **A**. Standardized effect sizes across 360 cortical regions showing X-chromosome dosage (XXY vs. XY) effects on CV, SA and CT **B**. Same as panel **A** but for Y-chromosome dosage effects (XYY vs. XY). **C.** Same as panel **A** but for testicular hormone production effects (male controls vs. males with IGD).

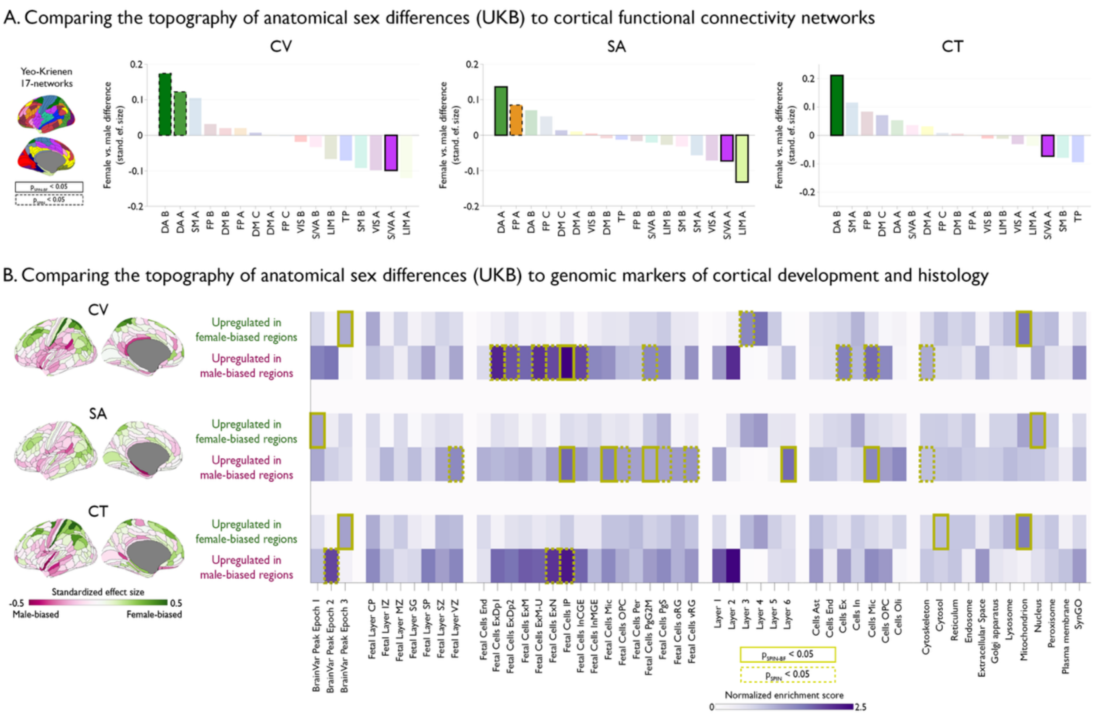

Supplementary Figure 4. Sex differences in cortical anatomy from the UKB cohort show varying enrichments for diverse functional networks and molecular signatures depending on their direction and whether they involve cortical volume (CV), surface area (SA) or thickness (CT). **A**. The mean standardized effect sizes of sex differences in each of the 17 Yeo-Krienen resting-state functional networks (left cortical map) were compared to spin-based spatial permutations of sex difference and functional network maps to determine two-tailed statistical significance. Bar plots quantity the mean neuroanatomical sex difference within each network for each of the 3 cortical features considered. Significant enrichments are shown in saturated bars with borders. Solid outlines survive Bonferroni correction across 3 cortical features ($p_{SPIN-BF}<0.05$), dashed outlines show nominal significance ($p_{SPIN}<0.05$). Bars are colored according to network designations shown in Yeo-Krienen network designations shown on the accompanying cortical map. SM: somatomotor, DA: dorsal attention, FP: frontoparietal, S/VA: salience/ventral attention/ LIM: limbic, DM: default mode, VIS: visual, TP: temporoparietal. **B.** Gene Set Enrichment Analysis[45] tests for genes with cortical expression patterns that correlate with the regional magnitude of female- and male-biased in CV, SA and CT. Normalized enrichment score for these annotations are shown in purple. Statistical significance is again shown at two thresholds (dashed boxes: nominal $p_{SPIN}<0.05$; solid boxes: Bonferroni-corrected $p_{SPIN-BF}<0.05$) as determined by

one-tailed test to assess whether the empirical p value is lower than the p values derived from spin-based spatial permutations of sex difference maps (Methods). The cortical maps show standardized effect sizes of sex differences in CV, SA and CT from **Figure 1A** as a reference. CP: cortical plate, IZ: intermediate zone, MZ: marginal zone, SG: supragranular, SP: subplate, VZ: ventricular zone, End: endothelial, ExDp: excitatory deep layer, ExM: excitatory maturing, ExN: excitatory migrating, IP: intermediate progenitor, InCGE: interneuron caudal ganglionic eminence, InMGE: interneuron medial ganglionic eminence, Mic: microglia, OPC: oligodendrocyte progenitor cell, Per: pericytes, PgG2M: cycling progenitor (G2- and M-phase), PgS: cycling progenitor (S-phase), oRG: outer radial glia, vRG: ventricular radial glia. Ast: astrocyte, Ex: excitatory, In: inhibitory, Oli: oligodendrocyte, SynGO: synaptic gene ontologies and annotations.

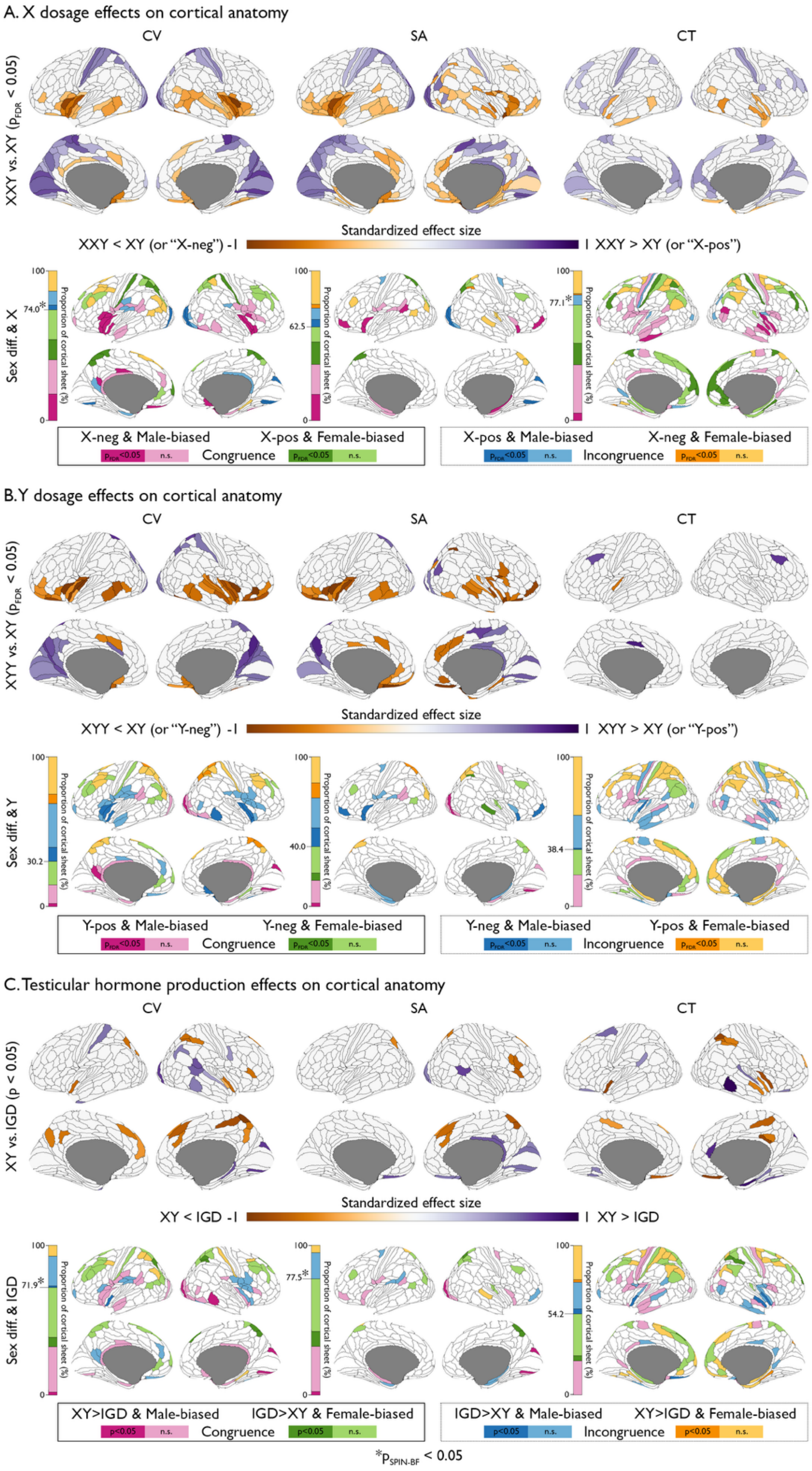
A. X dosage effects on cortical anatomy
CV
SA
CT
XXY vs. XY (p_{FDR} < 0.05)
Standardized effect size
XXY < XY (or "X-neg") -1
1 XXY > XY (or "X-pos")
Sex diff. & X
Proportion of cortical sheet (%)
100
74.0*
62.5
77.1*
0
X-neg & Male-biased
X-pos & Female-biased
Congruence
X-pos & Male-biased
X-neg & Female-biased
Incongruence
p_{FDR}<0.05
n.s.
B. Y dosage effects on cortical anatomy
CV
SA
CT
XYY vs. XY (p_{FDR} < 0.05)
Standardized effect size
XYY < XY (or "Y-neg") -1
1 XYY > XY (or "Y-pos")
Sex diff. & Y
Proportion of cortical sheet (%)
100
30.2
40.0
38.4
0
Y-pos & Male-biased
Y-neg & Female-biased
Congruence
Y-neg & Male-biased
Y-pos & Female-biased
Incongruence
p_{FDR}<0.05
n.s.
C. Testicular hormone production effects on cortical anatomy
CV
SA
CT
XY vs. IGD (p < 0.05)
Standardized effect size
XY < IGD -1
1 XY > IGD
Sex diff. & IGD
Proportion of cortical sheet (%)
100
71.9*
77.5*
54.2
0
XY>IGD & Male-biased
IGD>XY & Female-biased
Congruence
IGD>XY & Male-biased
XY>IGD & Female-biased
Incongruence
p<0.05
n.s.
*$p_{SPIN-BF}$ < 0.05

Supplementary Figure 5. Effects of sex chromosome dosage and testicular hormone production on regional cortical anatomy with analysis of overlaps with maps of sex-biased cortical anatomy in the UK Biobank. **A**. First row: standardized effect sizes across 360 cortical regions showing statistically significant (p_{FDR}<0.05) X dosage effects (i.e. XXY vs. XY differences) on CV, SA and CT using a two-tailed t-test after controlling for age, Euler and global phenotypes (total CV, total SA and total mean CT). Positive effect sizes (XXY > XY or "X-pos") are shown in purple, while negative effect sizes (XXY < XY or "X-neg") are shown in orange. Second row: region-wise congruence between the effects of sex differences from the UKB cohort and X dosage. Congruence is defined when the X dosage effect is significantly in congruent direction (darker) or insignificantly in congruent direction (lighter) for female- (green) and male-biased (pink) regions. Incongruence is defined as the effect showing significantly incongruent direction (darker) and insignificantly incongruent direction (lighter) for female- (orange) and male-biased (blue) regions. The bars show the proportions at which these conjunction cases make up the cortical sheet – providing numerical insets for those proportions which are statistically significantly larger (one-tailed, $p_{SPIN-BF}$<0.05) than expected under the null hypothesis based on 10,000 spatial permutations of sex difference maps. Asterisks indicate Bonferroni-corrected significance at 0.05 relative to spin-based spatial permutations of the HCP sex difference maps. **B**. Same as panel **A** but for statistically significant (p_{FDR}<0.05) Y-chromosome dosage effects (i.e. XYY vs. XY differences) based on a two-tailed t-test after controlling for the same covariates. **C**. Same as panel **A** but for statistically significant (p<0.05) effects of intact testicular hormone production (i.e. differences between male controls and males with IGD) based on a two-tailed t-test after controlling for the same covariates. Source data are provided as Source Data - Sex Differences in Human Cortical Anatomy.xlsx.
