# Supplementary material for: Regional sex differences in human cortical anatomy vary in their morphometric bases and overlap with sex chromosomal and gonadal influences
Source: Nat Commun. 2026 Jun 13;17:7503. doi: 10.1038/s41467-026-74274-8 (PMC13408908; doi:10.1038/s41467-026-74274-8)
Supplement: Supplementary file 2 — Reporting Summary [file 41467_2026_74274_MOESM2_ESM.pdf]

nature portfolio

Corresponding author(s): Hyo Lee, Armin Raznahan

Last updated by author(s): May 10, 2026

Reporting Summary

Nature Portfolio wishes to improve the reproducibility of the work that we publish. This form provides structure for consistency and transparency in reporting. For further information on Nature Portfolio policies, see our Editorial Policies and the Editorial Policy Checklist.

Statistics

For all statistical analyses, confirm that the following items are present in the figure legend, table legend, main text, or Methods section.

n/a	Confirmed	
☐	☒	The exact sample size (*n*) for each experimental group/condition, given as a discrete number and unit of measurement
☐	☒	A statement on whether measurements were taken from distinct samples or whether the same sample was measured repeatedly
☐	☒	The statistical test(s) used AND whether they are one- or two-sided<br>*Only common tests should be described solely by name; describe more complex techniques in the Methods section.*
☐	☒	A description of all covariates tested
☐	☒	A description of any assumptions or corrections, such as tests of normality and adjustment for multiple comparisons
☐	☒	A full description of the statistical parameters including central tendency (e.g. means) or other basic estimates (e.g. regression coefficient) AND variation (e.g. standard deviation) or associated estimates of uncertainty (e.g. confidence intervals)
☐	☒	For null hypothesis testing, the test statistic (e.g. *F*, *t*, *r*) with confidence intervals, effect sizes, degrees of freedom and *P* value noted<br>*Give P values as exact values whenever suitable.*
☒	☐	For Bayesian analysis, information on the choice of priors and Markov chain Monte Carlo settings
☒	☐	For hierarchical and complex designs, identification of the appropriate level for tests and full reporting of outcomes
☐	☒	Estimates of effect sizes (e.g. Cohen's *d*, Pearson's *r*), indicating how they were calculated

Our web collection on statistics for biologists contains articles on many of the points above.

Software and code

Policy information about availability of computer code

Data collection	No software was used for data collection.
Data analysis	FreeSurfer (7.1.0). Details of the data analyses, including primary software packages and mathematical models, are provided in the Methods section.

For manuscripts utilizing custom algorithms or software that are central to the research but not yet described in published literature, software must be made available to editors and reviewers. We strongly encourage code deposition in a community repository (e.g. GitHub). See the Nature Portfolio guidelines for submitting code & software for further information.

Data

Policy information about availability of data

All manuscripts must include a data availability statement. This statement should provide the following information, where applicable:

- Accession codes, unique identifiers, or web links for publicly available datasets
- A description of any restrictions on data availability
- For clinical datasets or third party data, please ensure that the statement adheres to our policy

Source Data - Sex Differences in Human Cortical Anatomy.xlsx contains: the group-level statistical parameters (standardized effect sizes, nominal p values and p values adjusted for multiple comparison) for the effects of sex, sex chromosome dosage, and testicular hormone production; the enrichments for resting-state functional networks; and the ranked gene lists for the gene set enrichment analysis of cortical sex difference maps. The HCP S1200 data release (March 2017) is publicly available at https://db.humanconnectome.org/. The UK Biobank data release (2020) is publicly available at https://www.ukbiobank.ac.uk. For the HCP and

UKB datasets, the data access agreements and privacy policies prohibit sharing participant-level data such as neuroimaging-derived variables.

Research involving human participants, their data, or biological material

Policy information about studies with human participants or human data. See also policy information about sex, gender (identity/presentation), and sexual orientation and race, ethnicity and racism.

Reporting on sex and gender	Sex was self-reported in the HCP dataset and incorporated into the study design, as our primary objective was to examine normative sex differences in human brain anatomy. Sex was determined based on individual's sex chromosomes (XX: females, XY, XXY and XYY: males) for the UKB and clinical (XXY, XYY and IGD) datasets. Disaggregated information on sex and gender was not collected in the datasets used in this study.
Reporting on race, ethnicity, or other socially relevant groupings	Healthy participants in the HCP dataset were recruited to broadly reflect the ethnic and racial composition of the U.S. population. Individuals with non-Hispanic European ancestry from the UK Biobank dataset were included in this study. The clinical XXY, XYY and IGD cohorts were mainly non-Hispanic white.
Population characteristics	From the HCP dataset, we studied 592 females (mean±std age = 29.56±3.6) and 493 males (age = 27.89±3.59). Although the groups differed slightly in mean age ($p<0.05$, two-sample t-test), all participants were within the same age range (22-37), and age was included as a covariate in the models to estimate sex differences. The mean age did not differ in all other datasets: UKB dataset in this study included 375 females (age = 48.75±0.94) and 294 males (age = 48.89±0.83); XXY dataset included 99 individuals with XXY (age = 16.38±4.78) and 92 XY controls (age = 16.24±5.64); XYY dataset included 34 individuals with XYY (age = 15.49±5.33) and 47 XY controls (age = 13.97±5.76); and IGD dataset included individuals with 19 IGD (age = 23.27 ±8.33) and 22 XY controls (age = 21.89±7.66). Age was used as a covariate in all models estimating sex differences, X-chromosome dosage, Y-chromosome dosage and testicular hormone production.
Recruitment	The primary HCP participant pool consisted of healthy individuals born in Missouri. That for UK Biobank consisted of healthy individuals born in United Kingdoms. Details on recruitment procedures for HCP and UKB datasets are available on https://db.humanconnectome.org/ and https://www.ukbiobank.ac.uk, respectively. For the XXY and XYY datasets, all participants were recruited through parent support organizations, the National Institute of Mental Health and the National Institutes of Health Healthy Volunteers office. For the IGD dataset, all participants were recruited through referrals from collaborators and local physicians or self-referrals with advertisement of the study on the NIH and Eunice Kennedy Shriver National Institute of Child Health and Human Development websites as well as ClinicalTrials.gov.
Ethics oversight	All analyses and the research protocol in this study were approved by the institutional review board at the National Institute of Mental Health.

Note that full information on the approval of the study protocol must also be provided in the manuscript.

Field-specific reporting

Please select the one below that is the best fit for your research. If you are not sure, read the appropriate sections before making your selection.

☒ Life sciences ☐ Behavioural & social sciences ☐ Ecological, evolutionary & environmental sciences

For a reference copy of the document with all sections, see nature.com/documents/nr-reporting-summary-flat.pdf

Life sciences study design

All studies must disclose on these points even when the disclosure is negative.

Sample size	The sample size was determined by the number of participants included in the HCP S1200 data release (March 2017) and UKB data release (2020). The sample sizes of clinical datasets were determined by the total number of MRI scans available.
Data exclusions	We excluded 8 participants from the HCP dataset with an Euler number below -217, a conservative threshold recommended by Rosen et al., (2018) to ensure high-quality surface reconstruction. For the UKB dataset, we excluded participants who were 50 years of age or older.
Replication	Replication for sex differences found in the HCP dataset was performed within the sample across varying sizes and in the UKB dataset of 669 individuals between age range of 44-49 years.
Randomization	Not applicable.
Blinding	Participants are grouped by self-reported sex (for HCP dataset) and sex chromosome complements (all other datasets)

Reporting for specific materials, systems and methods

We require information from authors about some types of materials, experimental systems and methods used in many studies. Here, indicate whether each material, system or method listed is relevant to your study. If you are not sure if a list item applies to your research, read the appropriate section before selecting a response.

Materials & experimental systems

n/a	Involved in the study
☐	☐ Antibodies
☐	☐ Eukaryotic cell lines
☐	☐ Palaeontology and archaeology
☐	☐ Animals and other organisms
☐	☒ Clinical data
☐	☐ Dual use research of concern
☐	☐ Plants

Methods

n/a	Involved in the study
☐	☐ ChIP-seq
☐	☐ Flow cytometry
☐	☒ MRI-based neuroimaging

Antibodies

Antibodies used

Describe all antibodies used in the study; as applicable, provide supplier name, catalog number, clone name, and lot number.

Validation

Describe the validation of each primary antibody for the species and application, noting any validation statements on the manufacturer's website, relevant citations, antibody profiles in online databases, or data provided in the manuscript.

Eukaryotic cell lines

Policy information about cell lines and Sex and Gender in Research

Cell line source(s)

State the source of each cell line used and the sex of all primary cell lines and cells derived from human participants or vertebrate models.

Authentication

Describe the authentication procedures for each cell line used OR declare that none of the cell lines used were authenticated.

Mycoplasma contamination

Confirm that all cell lines tested negative for mycoplasma contamination OR describe the results of the testing for mycoplasma contamination OR declare that the cell lines were not tested for mycoplasma contamination.

Commonly misidentified lines
(See ICLAC register)

Name any commonly misidentified cell lines used in the study and provide a rationale for their use.

Palaeontology and Archaeology

Specimen provenance

Provide provenance information for specimens and describe permits that were obtained for the work (including the name of the issuing authority, the date of issue, and any identifying information). Permits should encompass collection and, where applicable, export.

Specimen deposition

Indicate where the specimens have been deposited to permit free access by other researchers.

Dating methods

If new dates are provided, describe how they were obtained (e.g. collection, storage, sample pretreatment and measurement), where they were obtained (i.e. lab name), the calibration program and the protocol for quality assurance OR state that no new dates are provided.

☐ Tick this box to confirm that the raw and calibrated dates are available in the paper or in Supplementary Information.

Ethics oversight

Identify the organization(s) that approved or provided guidance on the study protocol, OR state that no ethical approval or guidance was required and explain why not.

Note that full information on the approval of the study protocol must also be provided in the manuscript.

Animals and other research organisms

Policy information about studies involving animals; ARRIVE guidelines recommended for reporting animal research, and Sex and Gender in Research

Laboratory animals

For laboratory animals, report species, strain and age OR state that the study did not involve laboratory animals.

Wild animals

Provide details on animals observed in or captured in the field; report species and age where possible. Describe how animals were caught and transported and what happened to captive animals after the study (if killed, explain why and describe method; if released, say where and when) OR state that the study did not involve wild animals.

Reporting on sex

Indicate if findings apply to only one sex; describe whether sex was considered in study design, methods used for assigning sex. Provide data disaggregated for sex where this information has been collected in the source data as appropriate; provide overall numbers in this Reporting Summary. Please state if this information has not been collected. Report sex-based analyses where performed, justify reasons for lack of sex-based analysis.

Field-collected samples	*For laboratory work with field-collected samples, describe all relevant parameters such as housing, maintenance, temperature, photoperiod and end-of-experiment protocol OR state that the study did not involve samples collected from the field.*
Ethics oversight	*Identify the organization(s) that approved or provided guidance on the study protocol, OR state that no ethical approval or guidance was required and explain why not.*

Note that full information on the approval of the study protocol must also be provided in the manuscript.

Clinical data

Policy information about clinical studies

All manuscripts should comply with the ICMJE guidelines for publication of clinical research and a completed CONSORT checklist must be included with all submissions.

Clinical trial registration	NCT00001246, NCT01500447
Study protocol	HCP and UKB data are de-identified and their usage does not fall under IRB review. The clinical datasets (XXY, XYY and IGD) are de-identified and their usage has been approved by IRB of the National Institute of Mental Health.
Data collection	Details are provided in https://pmc.ncbi.nlm.nih.gov/articles/PMC3724347/ (HCP) and https://pubmed.ncbi.nlm.nih.gov/25826379/ (UKB). For XXY and XYY datasets, details are available at https://www.clinicaltrials.gov/study/NCT00001246. For IGD dataset, details can be found at https://clinicaltrials.gov/study/NCT01500447.
Outcomes	Sex differences in cortical anatomy. X-chromosome dosage, Y-chromosome dosage and testicular hormone production effects on cortical anatomy.

Dual use research of concern

Policy information about dual use research of concern

Hazards

Could the accidental, deliberate or reckless misuse of agents or technologies generated in the work, or the application of information presented in the manuscript, pose a threat to:

No	Yes	
☒	☐	Public health
☒	☐	National security
☒	☐	Crops and/or livestock
☒	☐	Ecosystems
☒	☐	Any other significant area

Experiments of concern

Does the work involve any of these experiments of concern:

No	Yes	
☒	☐	Demonstrate how to render a vaccine ineffective
☒	☐	Confer resistance to therapeutically useful antibiotics or antiviral agents
☒	☐	Enhance the virulence of a pathogen or render a nonpathogen virulent
☒	☐	Increase transmissibility of a pathogen
☒	☐	Alter the host range of a pathogen
☒	☐	Enable evasion of diagnostic/detection modalities
☒	☐	Enable the weaponization of a biological agent or toxin
☒	☐	Any other potentially harmful combination of experiments and agents

Plants

Seed stocks	*Report on the source of all seed stocks or other plant material used. If applicable, state the seed stock centre and catalogue number. If plant specimens were collected from the field, describe the collection location, date and sampling procedures.*
Novel plant genotypes	*Describe the methods by which all novel plant genotypes were produced. This includes those generated by transgenic approaches, gene editing, chemical/radiation-based mutagenesis and hybridization. For transgenic lines, describe the transformation method, the number of independent lines analyzed and the generation upon which experiments were performed. For gene-edited lines, describe the editor used, the endogenous sequence targeted for editing, the targeting guide RNA sequence (if applicable) and how the editor was applied.*
Authentication	*Describe any authentication procedures for each seed stock used or novel genotype generated. Describe any experiments used to assess the effect of a mutation and, where applicable, how potential secondary effects (e.g. second site T-DNA insertions, mosiacism, off-target gene editing) were examined.*

ChIP-seq

Data deposition

☐ Confirm that both raw and final processed data have been deposited in a public database such as GEO.

☐ Confirm that you have deposited or provided access to graph files (e.g. BED files) for the called peaks.

Data access links<br>*May remain private before publication.*	*For "Initial submission" or "Revised version" documents, provide reviewer access links. For your "Final submission" document, provide a link to the deposited data.*
Files in database submission	*Provide a list of all files available in the database submission.*
Genome browser session<br>(e.g. UCSC)	*Provide a link to an anonymized genome browser session for "Initial submission" and "Revised version" documents only, to enable peer review. Write "no longer applicable" for "Final submission" documents.*

Methodology

Replicates	*Describe the experimental replicates, specifying number, type and replicate agreement.*
Sequencing depth	*Describe the sequencing depth for each experiment, providing the total number of reads, uniquely mapped reads, length of reads and whether they were paired- or single-end.*
Antibodies	*Describe the antibodies used for the ChIP-seq experiments; as applicable, provide supplier name, catalog number, clone name, and lot number.*
Peak calling parameters	*Specify the command line program and parameters used for read mapping and peak calling, including the ChIP, control and index files used.*
Data quality	*Describe the methods used to ensure data quality in full detail, including how many peaks are at FDR 5% and above 5-fold enrichment.*
Software	*Describe the software used to collect and analyze the ChIP-seq data. For custom code that has been deposited into a community repository, provide accession details.*

Flow Cytometry

Plots

Confirm that:

☐ The axis labels state the marker and fluorochrome used (e.g. CD4-FITC).

☐ The axis scales are clearly visible. Include numbers along axes only for bottom left plot of group (a 'group' is an analysis of identical markers).

☐ All plots are contour plots with outliers or pseudocolor plots.

☐ A numerical value for number of cells or percentage (with statistics) is provided.

Methodology

Sample preparation	*Describe the sample preparation, detailing the biological source of the cells and any tissue processing steps used.*
Instrument	*Identify the instrument used for data collection, specifying make and model number.*
Software	*Describe the software used to collect and analyze the flow cytometry data. For custom code that has been deposited into a community repository, provide accession details.*

Cell population abundance	*Describe the abundance of the relevant cell populations within post-sort fractions, providing details on the purity of the samples and how it was determined.*
Gating strategy	*Describe the gating strategy used for all relevant experiments, specifying the preliminary FSC/SSC gates of the starting cell population, indicating where boundaries between "positive" and "negative" staining cell populations are defined.*

☐ Tick this box to confirm that a figure exemplifying the gating strategy is provided in the Supplementary Information.

Magnetic resonance imaging

Experimental design

Design type	Structural MRI
Design specifications	Standard clinical MRI sessions including structural, diffusion MRI and functional MRI lasting approximately 1 hour.
Behavioral performance measures	N/A

Acquisition

Imaging type(s)	Structural
Field strength	3T
Sequence & imaging parameters	The HCP participants were scanned using 3T ConnectomeScanner (adapted from Siemens Skyra, Siemens Healthineer) with the following parameters: 0.7 mm isotropic resolution, FOV = 224 mm, TR = 2400 ms, TE = 2.14 ms, TI = 1000 ms, FA = 8°, bandwidth = 210 Hz per pixel, and GRAPPA = 2. The UKB participants were scanned using 3T Siemens Skyra with the following parameters: 1 mm isotropic resolution, FOV = 256 mm, TR = 2000 ms, TE = 2.01 ms, TI = 880 ms, FA = 8°, 240 Hz per pixel, echo spacing = 6.1 ms and GRAPPA = 2. The XXY and XYY cohorts were scanned using 3T MR750 scanner (General Electric) with the following parameters: 1 mm isotropic resolution, FOV = 256 mm, TR = 2530 ms, TE = 3.5ms, TI = 1100 ms, FA = 7°, 195.3 Hz per pixel and GRAPPA = 2. Individuals in the IGD cohort were scanned using 3T MR750 scanner (General Electric) with the following parameters: 1 mm isotropic resolution, FOV = 256 mm, TR = 3205 ms, TE1-4 = 1.888, 3.832, 5.776, 7.72 ms, TI = 1150 ms, FA = 7°, bandwidth = 651 Hz per pixel, and GRAPPA = 2.
Area of acquisition	Whole brain.

Diffusion MRI ☐ Used ☒ Not used

Preprocessing

Preprocessing software	FreeSurfer (7.1.0)
Normalization	Native MRI scans were spatially normalized to the MNI 152 volumetric template from which the cortical surfaces were reconstructed. Morphometric measures were then sampled on 360 regions defined by the HCP-MMP1.0 parcellation (Glasser et al., 2016).
Normalization template	MNI 152 volumetric template
Noise and artifact removal	N/A
Volume censoring	N/A

Statistical modeling & inference

Model type and settings	Euler index was used as a covariate for all statistical models in this study.
Effect(s) tested	Standardized effects of sex, X-chromosome dosage, Y-chromosome dosage and testicular hormone production.

Specify type of analysis: ☒ Whole brain ☐ ROI-based ☐ Both

Statistic type for inference (See Eklund et al. 2016)	Statistical comparisons were performed within each of the 360 regions defined by the HCP-MMP1.0 cortical parcellation.
Correction	False discovery rate correction was applied to adjust for multiple comparisons across 360 cortical regions.

Models & analysis

n/a	Involved in the study
☐	☐ Functional and/or effective connectivity
☐	☐ Graph analysis
☐	☒ Multivariate modeling or predictive analysis

Functional and/or effective connectivity	*Report the measures of dependence used and the model details (e.g. Pearson correlation, partial correlation, mutual information).*
Graph analysis	*Report the dependent variable and connectivity measure, specifying weighted graph or binarized graph, subject- or group-level, and the global and/or node summaries used (e.g. clustering coefficient, efficiency, etc.).*
Multivariate modeling and predictive analysis	We used linear models that included sex, age, Euler index and global phenotypes as independent variables and the regional cortical volume, area and thickness as the outcome variables.
