# Supplementary material for: Regional sex differences in human cortical anatomy vary in their morphometric bases and overlap with sex chromosomal and gonadal influences
Source: Nat Commun. 2026 Jun 13;17:7503. doi: 10.1038/s41467-026-74274-8 (PMC13408908; doi:10.1038/s41467-026-74274-8)
Supplement: Supplementary file 3 — Transparent Peer Review file [file 41467_2026_74274_MOESM3_ESM.pdf]

nature portfolio

Peer Review File

Regional sex differences in human cortical anatomy vary in their morphometric bases and overlap with sex chromosomal and gonadal influences

Corresponding Author: Dr Armin Raznahan

This file contains all reviewer reports in order by version, followed by all author rebuttals in order by version.

Version 0:

Reviewer comments:

Reviewer #1

(Remarks to the Author)

In this work, Lee and colleagues examined sex differences in cortical neuroanatomy, explored how those differences may be linked to functional and molecular features, and quantified the chromosomal and hormonal contributions to those differences. Major strengths include the use of several large datasets (including HCP and UK Biobank with generally healthy adults as well as one with individuals with sex chromosome aneuplodies and another with individuals with isolated gonadotropin-releasing hormone deficiency). Their analyses yield several important findings. First, although sex differences in cortical volume were more similar to those in surface area than those in cortical thickness, sex differences in volume in several regions were driven entirely by CT. Second, sex differences in volume, area, and thickness were largely reproducible across the HCP and UK Biobank datasets. Third, distinct functional networks exhibited varying levels of sex differences in neuroanatomy, with regions in dorsal attention and somatomotor networks generally larger in females, and regions in limbic, visual, and ventral attention generally larger in males. Fourth, sex differences in volume, area, and thickness are spatially coupled with distinct genes, which vary in expression in terms of developmental timing, brain layers, and cell types. Fifth, influences of X dosage on sex differences in neuroanatomy are evident in SA and CT, while influences of testicular hormone production are are evident in SA. The analyses presented are quite thorough and strictly controlled for multiple comparisons. Given the broad interest in this topic, I am sure this work will be of interest to many neuroscientists. Please find below some comments/suggestions to improve the suitability of this manuscript for publication. I'm signing my review and am happy to be contacted by the authors if they have any questions.
- Elvisha Dhamala

1. From my understanding, the analyses relating the anatomical sex differences maps to functional networks, gene expression patterns, X dosage effect, Y dosage effects, and testicular hormone production were only conducted using the maps derived from HCP. Although these analyses are already quite thorough, repeating these analyses using the maps derived from UK Biobank would further validate these findings and demonstrate that the observed relationships are generalizable.
2. Authors point out that the left hippocampus exhibited statistically significant sex differences in opposite directions in HCP vs UKB, and propose that this may be due to age effects on the structure and the 35.6 year disparity in mean age between the datasets. This is misleading as the authors used a subset of data from UKB with a mean age of 48-49 years, compared to the subset of data from HCP with a mean age of 27-30 years. Therefore, although age could potentially be driving this discrepancy, I'd recommend clarifying the age differences in the data used in these analyses.
3. The SCA and IGD samples are younger than the HCP (and UK Biobank) samples. A brief discussion about how this age difference may be influencing the reported results comparing neuroanatomical sex differences to dosage and hormone effects would be helpful. Is it possible that some of these overlaps may shift with age? (Also see comment #1).
4. Authors point that that experiental factors can contribute to and shape brain development, but do not elaborate on this. As the HCP and UK Biobank are both adult cohorts and thus have accumulated decades of gendered experiences, it would be helpful to more explicitly discuss how gender may be contributing to the sex differences observed here and acknowledge the limitation of attributing those differences solely to sex.
5. I'm not entirely convinced that the "soft" agreements (or disagreements) reported here are meaningfully different from noise. It would be helpful if the authors could further explain their reasoning for this designation and provide more context for

the interpretations.

Reviewer #2

(Remarks to the Author)
Summary

This manuscript presents a comprehensive and methodologically rigorous analysis of sex differences in human cortical anatomy. The authors move beyond cortical volume (CV) alone and disaggregate sex effects into two biologically distinct components—cortical surface area (SA) and cortical thickness (CT)—demonstrating that these dimensions show regionally dissociable patterns of sexual dimorphism and reflect distinct developmental and molecular processes. The study further integrates evidence from SCA conditions (XXY, XYY) and isolated GnRH deficiency (IGD) cohorts to probe chromosomal and hormonal contributions to regional brain structure.

Using large, well-powered normative and clinical cohorts, reproducibility testing, cross-dataset validation, functional network annotation, and transcriptomic mapping, the authors provide one of the most detailed and biologically grounded accounts to date of sex-biased cortical organization. The work is technically strong, analytically sophisticated, and addresses a longstanding gap in how sex differences in cortical anatomy are interpreted mechanistically.
Overall, this is a highly rigorous, conceptually integrated, and field-defining paper that should be accepted for publication.

Points for Clarification

Figure 4 would benefit from explicit explanation of purple vs orange coloring and a clearer definition of “X-pos” and “X-neg” to improve interpretability for non-specialist readers.

Because sex chromosome aneuploidies are often accompanied by hormonal differences, could the authors clarify whether gonadal function or hormone treatment status was evaluated in the SCA cohorts and how potential hormonal influences were accounted for?

The manuscript notes that networks showing sex-biased cortical organization (sensorimotor, dorsal and ventral attention, and limbic) have also been implicated in sex-biased psychiatric disorders. If space permits, it would be helpful to clarify whether the observed sex differences in cortical volume, thickness, and surface area are consistent with neuroimaging findings in those disorders, and whether there is evidence linking network-level anatomical differences to known sex differences in prevalence or clinical presentation.

Reviewer #3

(Remarks to the Author)
In this paper, the authors conducted computational analyses of cortical anatomy (obtained through structural MRI scans) to determine whether regional sex differences in cortical volumes arise from sex differences in surface area (SA) and/or cortical thickness (CT). They used two large cohorts of typically developing controls (Human Connectome Project, HCP, and the UK Biobank; combined 1754 individuals; 967 females) as well as case-control clinical cohorts (combined 313 individuals) of sex chromosome aneuploidy (SCA) and isolated gonadotropin-releasing hormone deficiency (IGD) to determine whether regional sex differences in these three variables (CV, SA, CT) are determined by variations in sex chromosome dosage and/or or sex hormones. They observed that sex differences in regional cortical volume reflect spatially dissociable alterations of cortical surface area and thickness. The sex differences were strongly correlated between CV and SA, moderately correlated between CV and CT but weakly correlated between SA and CT. Despite the general dominance of SA as a driver of sex differences in CV, a region-by-region analysis revealed several examples of CV sex differences entirely driven by CT, i.e., in the primary sensory cortex, auditory cortex, and paralimbic cortices. Female biased regions tended to localize to dorsal cortical regions and male-biased ones to ventral regions. Furthermore, they obtained good between and within sample reproducibility of these regional sex differences, but importantly it was observed that sex differences can still be highly unstable at sex-balanced sample sizes of 400 individuals (thus confirming the need for large cohorts when studying sex differences). They also correlated these sex differences with some functional (resting state) and molecular signatures (gene expression profiles) obtaining some spatial alignments. Finally using SCA and IGD datasets, they observed a tendency for regions of sex-biased anatomy to show congruent effects of varying X chromosome dosage and functioning of the HPG axis, consistent with data obtained in mouse models (i.e., the FCG mouse model).

This paper provides a nice in-depth analysis of sex differences in cortical volumes, confirming earlier studies showing such sex differences. Its originality lies in the fact they have attempted to determine the origin of these sex differences, i.e. whether they arise by sex differences in surface area or cortical thickness providing some form of mechanistic stratification of sex effects of cortical anatomy, as well as whether sex chromosome dosage and hormone levels contribute to sex differences in cortical anatomy.

Two main concerns:

First, the large difference in age ranges between the different cohorts: the HCP (aged 22-37 years) versus UK biobank (aged 44-50 years). This is acknowledged by the authors, but it needs some more attention and discussion, in particularly since

some of the female samples within the UKB might have already attained menopause or are at the least perimenopausal. This is particularly relevant when attempting to link sex differences in cortical anatomy with hormone levels. In the same line, in women, the number or pregnancies has also been linked to significant changes (in particularly decreases) in gray matter so this should be taken into consideration as well.

Likewise, the age ranges of the SCA and ICD samples are not much in line with the age ranges of the HCP and UKB samples. Overall, they are much younger, most of them still adolescent or young adult, and might not have yet reached adult cortical volumes. Puberty is an important period of synaptic pruning leading to decreases in gray matter. This is not being discussed in the paper.

Second concern: the SCA/IGD samples in general since SCA cannot be completely dissociated from variations in circulating hormone levels. For example, XXY males have lower testosterone levels than XY males. Furthermore, IGD if severe, i.e., they are born with a micropenis and undescended testes, will be supplemented with testosterone at very young age which will still affect the developing brain.

Furthermore, when referring to X chromosome dosage, it is too bad that they did not attempt to include XO individuals (Turner Syndrome which is quite common), although it cannot be entirely dissociated from variations in hormone levels as well (lower estrogen levels in general). In the same line, the paper is somewhat biased in the idea that any sex differences would be derived from sex differences in testosterone levels during development and no particular attention is given to potential feminizing effects of estrogens. So why did they not include female IGD individuals, it would have provided important information on the contribution of ovarian estrogens to cortical anatomy.

Reviewer #4

(Remarks to the Author)
This is an exciting study on an important topic of broad scientific and public interest. The authors use large imaging data bases from the Human Connectome Project to disaggregate two features of brain anatomy, surface area and cortical thickness, and determine how they drive sex differences in cortical volume. Using rigorous statistical approaches they demonstrated the importance of sample size and by comparing to the British BioBank provide information on the reliability of the findings across large data sets. Using other large data sets establishing resting state functional networks and transcriptomic atlases of the human cortex they map these onto the imaging analyses to provide first steps towards identifying the underlying biological functions of regions exhibiting sex differences. Lastly, they take the extraordinary step of comparing findings in sex chromosome and gonadal status normative individuals to men with an additional X or Y chromosome or having a genetic condition resulting in lower than normal androgen exposure for at the least the early developmental period. The manuscript is well written and the results clearly presented but the interest in the topic of sex differences in human cortex is so broad and of such fundamental importance due to both its clinical relevance and the potential for both mis- and over-interpretation, it will be of value to assure the findings are accessible to the expert and non-expert alike. Comments below are intended towards achieving that end.
1) The title undersells the findings by collapsing all the variables under the term “fractionation” which most people won’t know what it refers to. More explicitly stating something along the lines of “Disaggregating surface area, cortical thickness, sex chromosomes and hormones in the analysis of human cortical sex differences” would be a mouthful but more likely to draw interest.
2) The abstract is heavy on what was done and very light on what was found. A few sentences regarding the more impactful observations would make the manuscript more accessible.
3) Is there a generally accepted interpretation of the importance of surface area versus cortical thickness? Meaning is one a matter of more cells, wider spacing? Different myelination? As presented the terms are fairly abstract and unmoored from a biological reality that most people can grasp.
4) Related to this, would it be possible to provide a simple cartoon-like diagram that shows the relationship between SA, CT and CV to assist the non-expert?
5) If understanding Fig 1E correctly, it indicates that 58% of the regions (is that 58% of the 360 regions or 58% of the cortical sheet?) exhibit no significant sex differences in CV, SA or CT. If that is not the case please make clear what is, and if true, some discussion of those regions showing no sex differences would be of value to the reader
6) Line 451 – (59% of cortical regions sex-biased considering all 3 features vs. 29% considering CV alone) – is this consistent with Figure 1 or is Figure 1 being misinterpreted by this reviewer?
7) Discussion of the Results of Figure 1 notes bilateral sex differences in various regions, does this mean when the term “bilateral” is not used that there is a unilateral sex difference? Overall were there more bilateral than unilateral differences and was there any skewing by sex as to whether there is more of bilateral versus unilateral differences?
8) For data in Fig 2, do the authors have any speculation why the variance in CT is notably smaller than that for SA and CV – is this a measurement artifact or a biological reality?
9) The use of the Allen Brain Atlas for mapping transcriptional profiles onto regions with sex differences is compromised by the inclusion of only one female in the dataset. Just as we know that training AI on biased data results in biased AI, it would seem there is a high probability of the same happening here. This is especially true in light of this being post-mortem data and both the causes and course of death being highly influenced by gender. Studies by Nestler and others have found profound sex differences in postmortem human cortex in the context of major depressive disorder etc. More attention to this caveat and some strategy for avoiding the early introduction of bias to the analyses would be beneficial.
10) Along these same lines, what was the sex distribution of subjects used to create the Yeo-Krienen 17 resting-state functional networks? Could there be similar sources of bias here?

Minor Comments
1) Is the sex difference in SA a by-product of the difference in overall brain size?
2) What is “split-half sample size”
3) What was the mean age for those with sex chromosome aneuploidies and IGD compared to controls?
4) Line 497 – “statistically surprising tendency”, should this be “surprising statistical trend”?

Version 1:

Reviewer comments:

Reviewer #1

(Remarks to the Author)
Authors have addressed all of my concerns.

Reviewer #2

(Remarks to the Author)
The authors have done an excellent job addressing prior noted concerns. The revisions are clear, thorough, and responsive to the original feedback. I am satisfied with the current version of the manuscript and have no further comments. I recommend this manuscript for publication.

Reviewer #3

(Remarks to the Author)
The authors have addressed all my concerns in a satisfactorily manner.

Reviewer #4

(Remarks to the Author)
The authors have done an excellent job responding to both mine and other reviewers suggestions for improvement, being both gracious and responsive. I did note there was no response to the request from Rev 3 (?) to discuss the absence of XO females and would be curious to hear the authors views on whether these individuals would be informative as to potential feminizing effects of estrogen as suggested by the reviewer, but this is in no way necessary for the publication which in my view can be accepted as is

Open Access This Peer Review File is licensed under a Creative Commons Attribution 4.0 International License, which permits use, sharing, adaptation, distribution and reproduction in any medium or format, as long as you give appropriate credit to the original author(s) and the source, provide a link to the Creative Commons license, and indicate if changes were made.
In cases where reviewers are anonymous, credit should be given to 'Anonymous Referee' and the source.

The images or other third party material in this Peer Review File are included in the article’s Creative Commons license, unless indicated otherwise in a credit line to the material. If material is not included in the article’s Creative Commons license and your intended use is not permitted by statutory regulation or exceeds the permitted use, you will need to obtain permission directly from the copyright holder.
To view a copy of this license, visit https://creativecommons.org/licenses/by/4.0/

Reviewer 1

Comment 1

In this work, Lee and colleagues examined sex differences in cortical neuroanatomy, explored how those differences may be linked to functional and molecular features, and quantified the chromosomal and hormonal contributions to those differences. Major strengths include the use of several large datasets (including HCP and UK Biobank with generally healthy adults as well as one with individuals with sex chromosome aneuplodies and another with individuals with isolated gonadotropin-releasing hormone deficiency). Their analyses yield several important findings. First, although sex differences in cortical volume were more similar to those in surface area than those in cortical thickness, sex differences in volume in several regions were driven entirely by CT. Second, sex differences in volume, area, and thickness were largely reproducible across the HCP and UK Biobank datasets. Third, distinct functional networks exhibited varying levels of sex differences in neuroanatomy, with regions in dorsal attention and somatomotor networks generally larger in females, and regions in limbic, visual, and ventral attention generally larger in males. Fourth, sex differences in volume, area, and thickness are spatially coupled with distinct genes, which vary in expression in terms of developmental timing, brain layers, and cell types. Fifth, influences of X dosage on sex differences in neuroanatomy are evident in SA and CT, while influences of testicular hormone production are evident in SA. The analyses presented are quite thorough and strictly controlled for multiple comparisons. Given the broad interest in this topic, I am sure this work will be of interest to many neuroscientists. Please find below some comments/suggestions to improve the suitability of this manuscript for publication. I'm signing my review and am happy to be contacted by the authors if they have any questions.

- Elvisha Dhamala

Response 1

We thank Dr. Elvisha Dhamala for their positive appraisal of our work as well as their constructive and instructive comments that led us to substantially improve our manuscript. Please see below of our point-by-point responses.

Comment 2

From my understanding, the analyses relating the anatomical sex differences maps to functional networks, gene expression patterns, X dosage effect, Y dosage effects, and testicular hormone production were only conducted using the maps derived from HCP. Although these analyses are

already quite thorough, repeating these analyses using the maps derived from UK Biobank would further validate these findings and demonstrate that the observed relationships are generalizable.

Response 2

We thank Dr. Dhamala for this suggestion and have now included the requested analyses comparing maps of sex differences in the UK Biobank (UKB) to (i) functional networks and gene expression patterns (new **Sup Figure 4**) and (ii) the anatomical influences of X-chromosome dosage, Y-chromosome dosage and testicular hormone production (new **Sup Figure 5**). These analyses establish strong replicability of our main findings from the HCP dataset, including: i) the enrichments for dorsal attention, salience/ventral attention, limbic and somatomotor networks; ii) transcriptomic alignments and associated gene set enrichments for sex difference maps (median cross-annotation normalized enrichment score between HCP and UKB analyses Pearson correlation = 0.83); and iii) congruence between anatomical sex differences and the influences of X-chromosome dosage and testicular hormone production. The new text sections and figures detailing these analyses are reproduced below.

Results, lines 298-303: "We observed significantly male-biased anatomy within the limbic network for CV and SA and the sensory/ventral attention network for CT, alongside significantly female-biased anatomy within the dorsal attention network for all three anatomical phenotypes and within the sensorimotor network for CT. *These network enrichments were reproduced when using maps of sex differences in regional CV, SA and CT from the UK Biobank dataset (**Sup Figure 4A**).*"

Results, lines 332-337: "*These enrichments collectively showed good congruence between analyses in the HCP and UKB (median cross-annotation normalized enrichment score Pearson correlation = 0.83; **Sup Figure 4B**), and were strong for each direction of sex bias for all 3 cortical anatomy phenotypes examined (Pearson correlation range = 0.71-0.86) with the isolated exception of enrichments for female-biased CT (R=-0.28).*"

Results, lines 446-451: "*Repeating these analytic procedures for the UKB dataset map of sex-biased anatomy replicated our observations: significant proportions of cortical regions showed congruence between sex differences and X effects on CV (73.5%) and CT (76.7%), as well as T effects on CV (72.5%) and SA (75.6%), with highly consistent sets of regions showing these congruences between analyses using HCP and UKB data (**Sup Figure 5**).*"

Supplementary Figure 4:

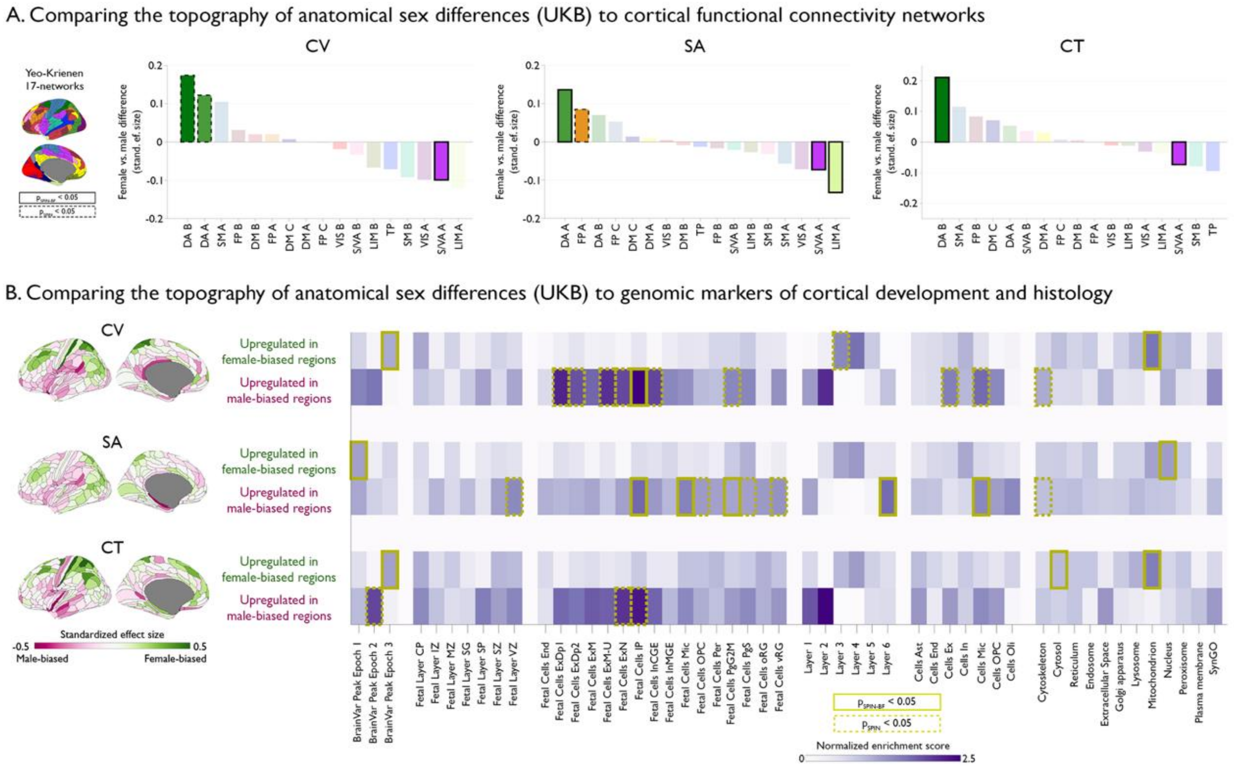

Supplementary Figure 4. Sex differences in cortical anatomy from the UKB cohort show varying enrichments for diverse functional networks and molecular signatures depending on their direction and whether they involve cortical volume (CV), surface area (SA) or thickness (CT). **A**. The mean standardized effect sizes of sex differences in each of the 17 Yeo-Krienen resting-state functional networks (left cortical map) were compared to spin-based spatial permutations of sex difference and functional network maps to determine statistical significance. Bar plots quantity the mean neuroanatomical sex difference within each network for each of the 3 cortical features considered. Significant enrichments are shown in saturated bars with borders. Solid outlines survive Bonferroni correction across 3 cortical features ($p_{SPIN-BF}<0.05$), dashed outlines show nominal significance ($p_{SPIN}<0.05$). Bars are colored according to network designations shown in Yeo-Krienen network designations shown on the accompanying cortical map. SM: somatomotor, DA: dorsal attention, FP: frontoparietal, S/VA: salience/ventral attention/ LIM: limbic, DM: default mode, VIS: visual, TP: temporoparietal. **B**. Gene Set Enrichment Analysis tests for genes with cortical expression patterns that correlate with the regional magnitude of female- and male-biased in CV, SA and CT. Statistical significance is again shown at two thresholds (nominal $p_{SPIN}<0.05$ and Bonferroni-corrected $p_{SPIN-BF}<0.05$) as derived from spin-based spatial permutation tests (**Methods**). The cortical maps show standardized effect sizes of sex differences in CV, SA and CT from **Figure 1A** as a reference. CP: cortical plate, IZ: intermediate zone, MZ: marginal zone, SG: supragranular, SP: subplate, VZ: ventricular zone, End: endothelial, ExDp: excitatory deep layer, ExM: excitatory maturing, ExN: excitatory migrating, IP: intermediate progenitor, InCGE: interneuron caudal ganglionic eminence, InMGE: interneuron medial ganglionic eminence, Mic: microglia, OPC: oligodendrocyte progenitor cell, Per: pericytes, PgG2M: cycling progenitor (G2- and M-phase), PgS: cycling progenitor (S-phase), oRG: outer radial glia, vRG: ventricular radial glia. Ast: astrocyte, Ex: excitatory, In: inhibitory, Oli: oligodendrocyte, SynGO: synaptic gene ontologies and annotations.

Supplementary Figure 5:

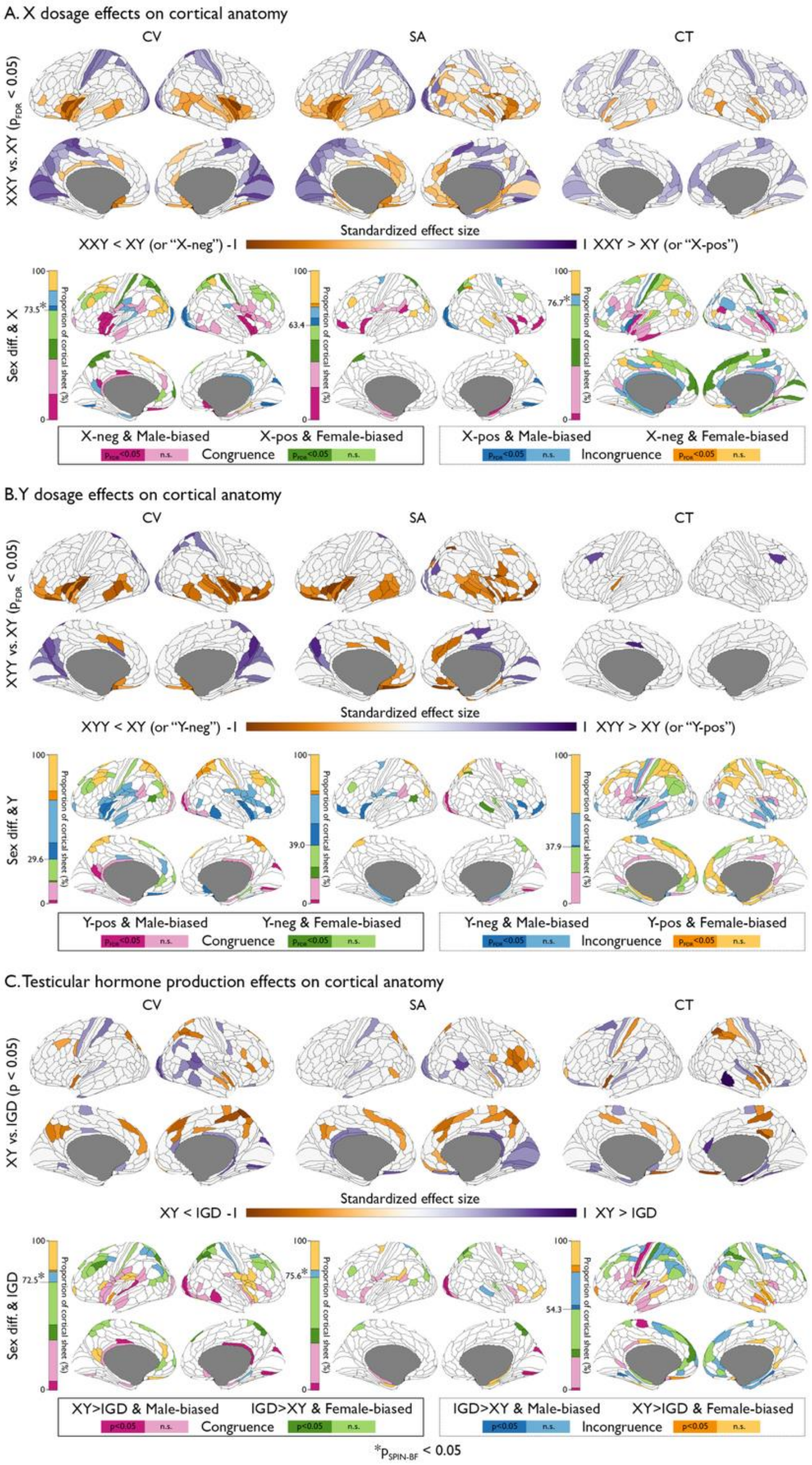

Supplementary Figure 5. Effects of sex chromosome dosage and testicular hormone production on regional cortical anatomy with analysis of overlaps with maps of sex-biased cortical anatomy in the UK Biobank. **A**. First row: standardized effect sizes across 360 cortical regions showing statistically significant (p_{FDR}<0.05) X-chromosome dosage effects (i.e. XXY vs. XY differences) on CV, SA and CT after controlling for age, Euler and global phenotypes (total CV, total SA and total mean CT). Positive effect sizes (XXY > XY or "X-pos") are shown in purple, while negative effect sizes (XXY < XY or "X-neg") are shown in orange. Second row: region-wise congruence between the effects of sex differences from the UKB cohort and X-chromosome dosage. Congruence is defined when the X-chromosome dosage effect is significantly in congruent direction (darker) or insignificantly in congruent direction (lighter) for female- (green) and male-biased (pink) regions. Incongruence is defined as the effect showing significantly incongruent direction (darker) and insignificantly incongruent direction (lighter) for female- (orange) and male-biased (blue) regions. The bars show the proportions at which these conjunction cases make up the cortical sheet – providing numerical insets for those proportions which are statistically significantly larger than expected under the null hypothesis. Asterisks indicate Bonferroni-corrected significance at 0.05 relative to spin-based spatial permutations of the HCP sex difference maps. **B**. Same as panel **A** but for statistically significant (p_{FDR}<0.05) Y-chromosome dosage effects (i.e. XYY vs. XY differences). **C**. Same as panel **A** but for statistically significant (p<0.05) effects of intact testicular hormone production (i.e. differences between male controls and males with IGD).

> Methods, lines 849-859: "***Replication of functional network, gene expression, sex chromosomal and gonadal comparisons with HCP sex differences using the UK Biobank dataset***
>
> *To evaluate the generalizability of our multiscale, sex chromosomal and gonadal findings, we used identical analytic procedures to repeat the functional networks and transcriptomic annotations and the sex chromosomal and gonadal congruence analyses using the sex difference maps derived from the UKB dataset. We then assessed if the functional networks that are anatomically sex-biased in the HCP analysis were preserved in the UKB analysis and if the normalized enrichment scores across transcriptomic annotations are similar between the HCP and UKB results. Moreover, we assessed if the sex chromosome dosage and testicular hormone production effects that show congruence in significant proportions of the cortical sheet, as well as the individual regions involved, are maintained between HCP and UKB datasets."*

Comment 3

Authors point out that the left hippocampus exhibited statistically significant sex differences in opposite directions in HCP vs UKB, and propose that this may be due to age effects on the structure and the 35.6 year disparity in mean age between the datasets. This is misleading as the authors used a subset of data from UKB with a mean age of 48-49 years, compared to the subset of data from HCP with a mean age of 27-30 years. Therefore, although age could potentially be driving this discrepancy, I'd recommend clarifying the age differences in the data used in these analyses.

Response 3

We thank Dr. Dhamala for catching this error. The mean age difference between HCP and the UKB subset used in this study is 20.01 years. We have corrected the age difference in the Results as below:

Results, line 278-279: "…and the *20-year* disparity in the mean age between HCP and UKB cohorts."

Comment 4

The SCA and IGD samples are younger than the HCP (and UK Biobank) samples. A brief discussion about how this age difference may be influencing the reported results comparing neuroanatomical sex differences to dosage and hormone effects would be helpful. Is it possible that some of these overlaps may shift with age? (Also see comment #1).

Response 4

We have now added new text to the Discussion section of our manuscript that highlights these age differences – noting that we observe spatial congruence of neuroanatomical sex-differences with sex chromosomal and testicular effects despite them. We now also underline the need to reassess the overlaps we report at different ages as suitable neuroimaging datasets in SCA and IGD become available. These manuscript changes are reproduced below.

Discussions, lines 560-567: "*These convergences are all the more striking when one considers the age differences between those samples we use to estimate sex chromosome and gonadal effects (spanning pre-puberty to young adulthood) and those in which we estimate sex differences in cortical anatomy (spanning young to late adulthood). Given the likely developmental dynamism of gonadal effects on brain anatomy and the theoretical potential for dynamism in sex chromosome effects*[1-10]*, it will be important to repeat our analyses when neuroimaging datasets on relatively rare SCA and IGD cases have become sufficiently large to allow age-stratified analyses.*"

Comment 5

Authors point that that experiential factors can contribute to and shape brain development, but do not elaborate on this. As the HCP and UK Biobank are both adult cohorts and thus have accumulated decades of gendered experiences, it would be helpful to more explicitly discuss how gender may be contributing to the sex differences observed here and acknowledge the limitation of attributing those differences solely to sex.

Response 5

We very much agree with the points made here and have now expanded the sections of our Discussion text that mention the importance of gendered experiences as shown below.

<u>Discussions, lines 571-579</u>: "*Although the present study has focused on chromosomal and gonadal factors, these are just some of the multitude of factors that could be contributing to observed sex differences in brain anatomy. In particular, neuroanatomical differences associated with sex may also be shaped by the many experiential factors that differ in their prevalence and form between females and males – including gender-related variables such as early socialization and gender roles*[11]*, educational and occupational choices*[12]*, gendered stressors*[13] *and cultural contexts*[14]*. Sex and gender are distinct but intertwined constructs that are especially hard to experimentally disambiguate in humans. Addressing this challenge will ultimately depend on integrating across a plurality of complementary research approaches and conceptual models.*"

Comment 6

I'm not entirely convinced that the "soft" agreements (or disagreements) reported here are meaningfully different from noise. It would be helpful if the authors could further explain their reasoning for this designation and provide more context for the interpretations.

Response 6

We appreciate this concern and agree that it is important to distinguish biologically meaningful findings from noise. We used "soft" to designate overlaps where both entities aligned (or opposed) in direction, but one was not statistically distinguishable from zero at $p_{FDR}<0.05$ cut off. This approach recognizes that statistical maps have spatial smoothness, and the definition of regions that are statistically significant is sensitive to the thresholds applied (which entail a degree of arbitrariness), the approach taken to correct for multiple comparisons and the sample size at which a given effect size is studied. Presenting "soft" overlaps provides more complete spatial coverage and – crucially – does not bias towards discovery of soft agreements vs. soft disagreements.

Reviewer 2

Comment 1

This manuscript presents a comprehensive and methodologically rigorous analysis of sex differences in human cortical anatomy. The authors move beyond cortical volume (CV) alone and disaggregate sex effects into two biologically distinct components—cortical surface area (SA) and cortical thickness (CT)—demonstrating that these dimensions show regionally dissociable patterns of sexual dimorphism and reflect distinct developmental and molecular processes. The study further integrates evidence from SCA conditions (XXY, XYY) and isolated GnRH deficiency (IGD) cohorts to probe chromosomal and hormonal contributions to regional brain structure.

Using large, well-powered normative and clinical cohorts, reproducibility testing, cross-dataset validation, functional network annotation, and transcriptomic mapping, the authors provide one of the most detailed and biologically grounded accounts to date of sex-biased cortical organization. The work is technically strong, analytically sophisticated, and addresses a longstanding gap in how sex differences in cortical anatomy are interpreted mechanistically.

Overall, this is a highly rigorous, conceptually integrated, and field-defining paper that should be accepted for publication.

Response 1

We are grateful to the reviewer for their appreciation of our work and for their constructive comments, which we have addressed as detailed below.

Comment 2

Figure 4 would benefit from explicit explanation of purple vs orange coloring and a clearer definition of "X-pos" and "X-neg" to improve interpretability for non-specialist readers.

Response 2

We thank the reviewer for this suggestion and have now modified the purple-orange color bar labels and added a sentence in the caption in Figure 4 and Supplemental Figure 5 to more clearly convey purple vs orange coloring and what "X-pos" and "X-neg" mean.

Figure 4:

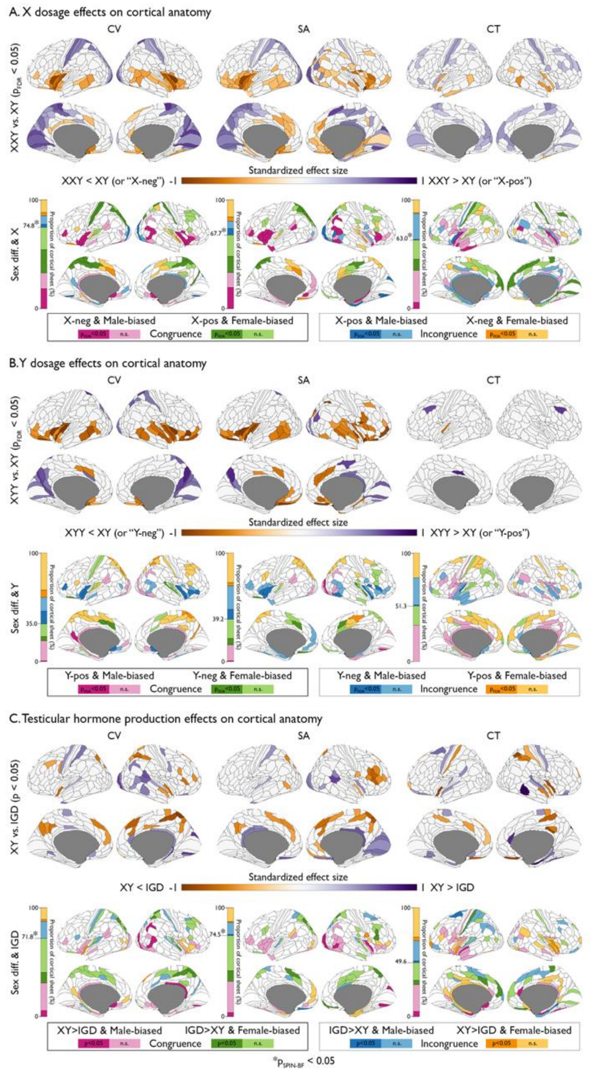

Supplementary Figure 5:

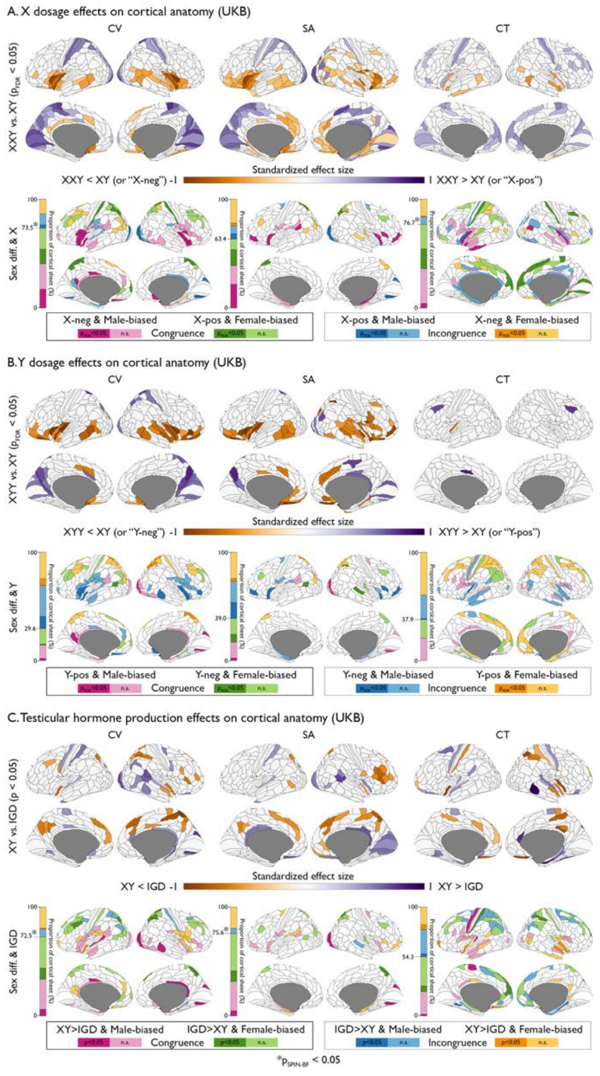

Figure 4 Caption, lines 407-409: "*Positive effect sizes (XXY > XY or "X-pos") are shown in purple, while negative effect sizes (XXY < XY or "X-neg") are shown in orange.*"

Supplementary Figure 5 Caption, lines 99-100: "*Positive effect sizes (XXY > XY or "X-pos") are shown in purple, while negative effect sizes (XXY < XY or "X-neg") are shown in orange.*"

Comment 3

Because sex chromosome aneuploidies are often accompanied by hormonal differences, could the authors clarify whether gonadal function or hormone treatment status was evaluated in the SCA cohorts and how potential hormonal influences were accounted for?

Response 3

We thank the reviewer for raising this important point. It is true that XXY syndrome is associated with hypogonadism, which could theoretically contribute to associated neuroanatomical changes. However, comparative studies across a wide range of X-chromosome dosage variation states indicate that the accompanying anatomical changes are seen irrespective of whether the modelled disparity in X-chromosome dosage is associated with male hypogonadism or not – suggesting that the anatomical effects are primarily reflective of direct X-chromosome dosage effects on the brain. We have now added new text to our manuscript that clarifies this for readers. We have also expanded sections of our Discussion section that underline the need for more studies of endocrine effects on regional brain anatomy in humans.

Results, lines 383-388: "*While XXY syndrome is associated with endocrine changes and treatments that could potentially contribute to reported alterations of cortical anatomy, prior work has shown that changes in regional anatomy with supernumerary X-chromosomes is consistent amongst multiple SCAs irrespective of their gonadal status (i.e. XXX, XXXX, XXYY, XXXXY)*[15] *and mirrored in X-monosomy*[16] *– suggesting that these anatomical effects are primarily reflective of direct sex chromosome dosage influences on the brain.*"

Discussion, lines 526-535: "It should be underlined however that SCAs and IGD are each complex medical conditions and therefore necessarily provide imperfect experimental probes for sex chromosomal and gonadal effects. Specifically, the effects of sex chromosome dosage variation in aneuploidy may not recapitulate those that operate in the euploidic context between XX and XY individuals, *and SCAs often feature abnormal gonadal function and variable hormone replacement regimens. These endocrine components of SCAs were not empirically addressed in our study and are an important area for future neuroimaging research – although reported changes of CV, SA and CT in SCAs appear to be primarily reflective of direct sex chromosome dosage effects rather than being modulated by gonadal context*[15,16]."

Comment 4

The manuscript notes that networks showing sex-biased cortical organization (sensorimotor, dorsal and ventral attention, and limbic) have also been implicated in sex-biased psychiatric disorders. If space permits, it would be helpful to clarify whether the observed sex differences in cortical volume, thickness, and surface area are consistent with neuroimaging findings in those disorders, and whether there is evidence linking network-level anatomical differences to known sex differences in prevalence or clinical presentation.

Response 4

We have now modified this section of our Discussion to provide more regional and phenotypic specifications regarding reported neuroanatomical alterations in regions of sex-biased cortical anatomy.

Discussion, lines 500-506: "Given this logic, it is notable that those resting-state functional connectivity networks, which show enrichment for anatomical sex-biases (sensorimotor, dorsal and ventral attention and limbic), have also been reported to show sex differences in their spatial distribution[17] and connectivity[18]. *Some of these networks – especially ventral attentional systems encompassing male-biased medial prefrontal and insula regions - have been reported to show recurrent anatomical reductions across multiple neuropsychiatric disorders including female-biased mood disorders*[19,20]."

Reviewer 3

Comment 1

In this paper, the authors conducted computational analyses of cortical anatomy (obtained through structural MRI scans) to determine whether regional sex differences in cortical volumes arise from sex differences in surface area (SA) and/or cortical thickness (CT). They used two large cohorts of typically developing controls (Human Connectome Project, HCP, and the UK Biobank; combined 1754 individuals; 967 females) as well as case-control clinical cohorts (combined 313 individuals) of sex chromosome aneuploidy (SCA) and isolated gonadotropin-releasing hormone deficiency (IGD) to determine whether regional sex differences in these three variables (CV, SA, CT) are determined by variations in sex chromosome dosage and/or or sex hormones. They observed that sex differences in regional cortical volume reflect spatially dissociable alterations of cortical surface area and thickness. The sex differences were strongly correlated between CV and SA, moderately correlated between CV and CT but weakly correlated between SA and CT. Despite the general dominance of SA as a driver of sex differences in CV, a region-by-region analysis revealed several examples of CV sex differences entirely driven by CT, i.e., in the primary sensory cortex, auditory cortex, and paralimbic cortices. Female biased regions tended to localize to dorsal cortical regions and male-biased ones to ventral regions. Furthermore, they obtained good between and within sample reproducibility of these regional sex differences, but importantly it was observed that sex differences can still be highly unstable at sex-balanced sample sizes of 400 individuals (thus confirming the need for large cohorts when studying sex differences). They also correlated these sex differences with some functional (resting state) and molecular signatures (gene expression profiles) obtaining some spatial alignments. Finally using SCA and IGD datasets, they observed a tendency for regions of sex-biased anatomy to show congruent effects of varying X chromosome dosage and functioning of the HPG axis, consistent with data obtained in mouse models (i.e., the FCG mouse model).

This paper provides a nice in-depth analysis of sex differences in cortical volumes, confirming earlier studies showing such sex differences. Its originality lies in the fact they have attempted to determine the origin of these sex differences, i.e. whether they arise by sex differences in surface area or cortical thickness providing some form of mechanistic stratification of sex effects of cortical anatomy, as well as whether sex chromosome dosage and hormone levels contribute to sex differences in cortical anatomy.

Response 1

We thank the reviewer for their in-depth and positive appraisal of our work and for raising crucial points that led us to substantial improvements in the interpretation of our findings.

On a side note, upon reading the reviewer's comment, we noticed that we had a typographical error in the Discussions concerning the sex-balanced sample size at which the anatomical sex differences can be unstable. What we meant to convey was that the sex differences can still be highly unstable at sample sizes of 200 individuals. We have corrected this error in the Discussions as below:

Discussions, line 490-491: "… the discovered topography of sex differences can still be highly unstable at sex-balanced sample sizes of *200 individuals*."

Please see below for our point-by-point responses to the reviewer's comments.

Comment 2

First, the large difference in age ranges between the different cohorts: the HCP (aged 22-37 years) versus UK biobank (aged 44-50 years). This is acknowledged by the authors, but it needs some more attention and discussion, in particularly since some of the female samples within the UKB might have already attained menopause or are at the least perimenopausal. This is particularly relevant when attempting to link sex differences in cortical anatomy with hormone levels. In the same line, in women, the number or pregnancies has also been linked to significant changes (in particularly decreases) in gray matter so this should be taken into consideration as well.

Likewise, the age ranges of the SCA and ICD samples are not much in line with the age ranges of the HCP and UKB samples. Overall, they are much younger, most of them still adolescent or young adult, and might not have yet reached adult cortical volumes. Puberty is an important period of synaptic pruning leading to decreases in gray matter. This is not being discussed in the paper.

Second concern: the SCA/IGD samples in general since SCA cannot be completely dissociated from variations in circulating hormone levels. For example, XXY males have lower testosterone levels than XY males. Furthermore, IGD if severe, i.e., they are born with a micropenis and undescended testes, will be supplemented with testosterone at very young age which will still affect the developing brain.

Furthermore, when referring to X chromosome dosage, it is too bad that they did not attempt to include XO individuals (Turner Syndrome which is quite common), although it cannot be entirely dissociated from variations in hormone levels as well (lower estrogen levels in general). In the same line, the paper is somewhat biased in the idea that any sex differences would be derived from sex differences in testosterone levels during development and no particular attention is given to potential feminizing effects of estrogens. So why did they not include female IGD individuals, it would have provided important information on the contribution of ovarian estrogens to cortical anatomy.

Response 2

We thank the reviewer for the many well-made points above. These points all concern limitations and caveats in use of SCA and male IGDs as models of sex chromosomal and gonadal influences on brain anatomy, which may be further compounded by mismatches between the age range when these conditions are studied compared to the age range when sex effects are mapped. Because of this thematic connection between these comments, we have addressed them through multiple changes (italicized) to the two relevant paragraphs of our Discussion as detailed below.

Discussion, lines 526-545: "*It should be underlined however that SCAs and IGD are each complex medical conditions and therefore necessarily provide imperfect experimental probes for sex chromosomal and gonadal effects.* Specifically, the effects of sex chromosome dosage variation in aneuploidy may not recapitulate those that operate in the euploidic context between XX and XY individuals, *and SCAs often feature abnormal gonadal function and variable hormone replacement regimens. These endocrine components of SCAs were not empirically addressed in our study and are an important area for future neuroimaging research – although reported changes of CV, SA and CT in SCAs appear to be primarily reflective of direct sex chromosome dosage effects rather than being modulated by gonadal context[15,16]. Similarly,* while IGD is a powerful model for gonadal insufficiency, it is also complicated by phenotypic variations in severity of hormone deficiency, heterogeneous hormonal replacement regimens and the potential for primary alterations in neurodevelopment[21]. *Furthermore, the extreme rarity of IGD in females prevented us from harnessing this model to probe important hypotheses regarding ovarian influences on brain anatomy. Addressing these limitations will ultimately hinge on accruing larger and better characterized neuroimaging samples of these relatively rare SCA and IGD conditions, as well as incorporating additional lenses on sex-chromosomal (e.g. through statical genetic analyses[22]) and gonadal* effects *on the brain (e.g. through neuroimaging studies of puberty[23], the menstrual cycle[24-26], pregnancy[27,28] and menopause[29] as well as andropause and other variations in male gonadal function[30,31]*."

Discussion, lines 546-547: "*Nevertheless, despite the many caveats above, our analyses in SCA and IGD reveal a surprising statistical trend for* regions of sex-biased anatomy to show congruent effects of varying X-chromosome dosage and hypothalamic-pituitary-gonadal axis function – consistent with the hypothesis that these sources of sex-biased biology may shape neuroanatomical sex differences in humans[32] as they have been experimentally shown to do in rodents[33]."

Discussion, lines 560-567: "*These convergences are all the more striking when one considers the age differences between those samples we use to estimate sex chromosome and gonadal effects (spanning pre-puberty to young adulthood) and those in which we estimate sex differences in cortical anatomy (spanning young to late adulthood). Given the likely developmental dynamism of gonadal effects on brain anatomy and the theoretical potential for dynamism in sex chromosome effects*[1-10]*, it will be important to repeat our analyses when neuroimaging datasets on relatively rare SCA and IGD cases have become sufficiently large to allow age-stratified analyses.*"

Reviewer 4

Comment 1

This is an exciting study on an important topic of broad scientific and public interest. The authors use large imaging data bases from the Human Connectome Project to disaggregate two features of brain anatomy, surface area and cortical thickness, and determine how they drive sex differences in cortical volume. Using rigorous statistical approaches they demonstrated the importance of sample size and by comparing to the British BioBank provide information on the reliability of the findings across large data sets. Using other large data sets establishing resting state functional networks and transcriptomic atlases of the human cortex they map these onto the imaging analyses to provide first steps towards identifying the underlying biological functions of regions exhibiting sex differences. Lastly, they take the extraordinary step of comparing findings in sex chromosome and gonadal status normative individuals to men with an additional X or Y chromosome or having a genetic condition resulting in lower than normal androgen exposure for at the least the early developmental period. The manuscript is well written and the results clearly presented but the interest in the topic of sex differences in human cortex is so broad and of such fundamental importance due to both its clinical relevance and the potential for both mis- and over-interpretation, it will be of value to assure the findings are accessible to the expert and non-expert alike. Comments below are intended towards achieving that end.

Response 1

We thank the reviewer for their appreciation and enthusiasm for our work as well as their constructive and insightful comments – which we have addressed as detailed below.

Comment 2

The title undersells the findings by collapsing all the variables under the term "fractionation" which most people won't know what it refers to. More explicitly stating something along the lines of "Disaggregating surface area, cortical thickness, sex chromosomes and hormones in the analysis of human cortical sex differences" would be a mouthful but more likely to draw interest.

Response 2

Many thanks for this suggestion. We have now changed the manuscript title to:

"Regional sex differences in human cortical anatomy vary in their morphometric bases and overlap with sex chromosomal and gonadal influences"

Comment 3

The abstract is heavy on what was done and very light on what was found. A few sentences regarding the more impactful observations would make the manuscript more accessible.

Response 3

We very much agree with this suggestion. Unfortunately, our current abstract word count (250) is already over the typical limit for Nature Communications (150) – making it hard to add more material. At the same time, the multi-component nature of our study design makes it hard to remove material because we need to orient readers to each element, including: mapping sex differences in CV; partitioning them in to SA and CT; annotating CV, SA and CT maps against both functional networks and gene expression gradients; mapping sex chromosome and gonadal effects on regional cortical anatomy; and, integrating these effects with sex differences.

Comment 4

Is there a generally accepted interpretation of the importance of surface area versus cortical thickness? Meaning is one a matter of more cells, wider spacing? Different myelination? As presented the terms are fairly abstract and unmoored from a biological reality that most people can grasp.

Response 4

We thank the reviewer for raising these points and have now added new text to the Discussion section of our manuscript that better considers surface area and thickness findings relative to their potential histological bases.

<u>Discussion, lines 473-481</u>: "This regional fractionation of CV sex differences according to the relative contribution of SA and CT provides a form of mechanistic stratification of sex effects of cortical anatomy. *Specifically, SA and CT are known to reflect largely distinct genetic*[34-36]*, cellular*[37,38] *and developmental*[1] *processes – with SA suggested to reflect the formation of radial columnar units and proliferation of progenitor cells in the ventricular zone during early prenatal development and CT suggested to reflect dendritic arborization, axonal density, myelination and glial support in these cortical columns during peri- and post-natal development.* Accordingly, these distinct biological processes would be predicted to differentially contribute to sex differences in cortical anatomy for regions of SA- vs. CT-driven sex-biased in CV."

Comment 5

Related to this, would it be possible to provide a simple cartoon-like diagram that shows the relationship between SA, CT and CV to assist the non-expert?

Response 5

We thank the reviewer for this suggestion and have included a diagram that conveys what CV, SA and CT represent in the 3D anatomical MRI space into Figure 1 and an explanation in the caption.

Figure 1:

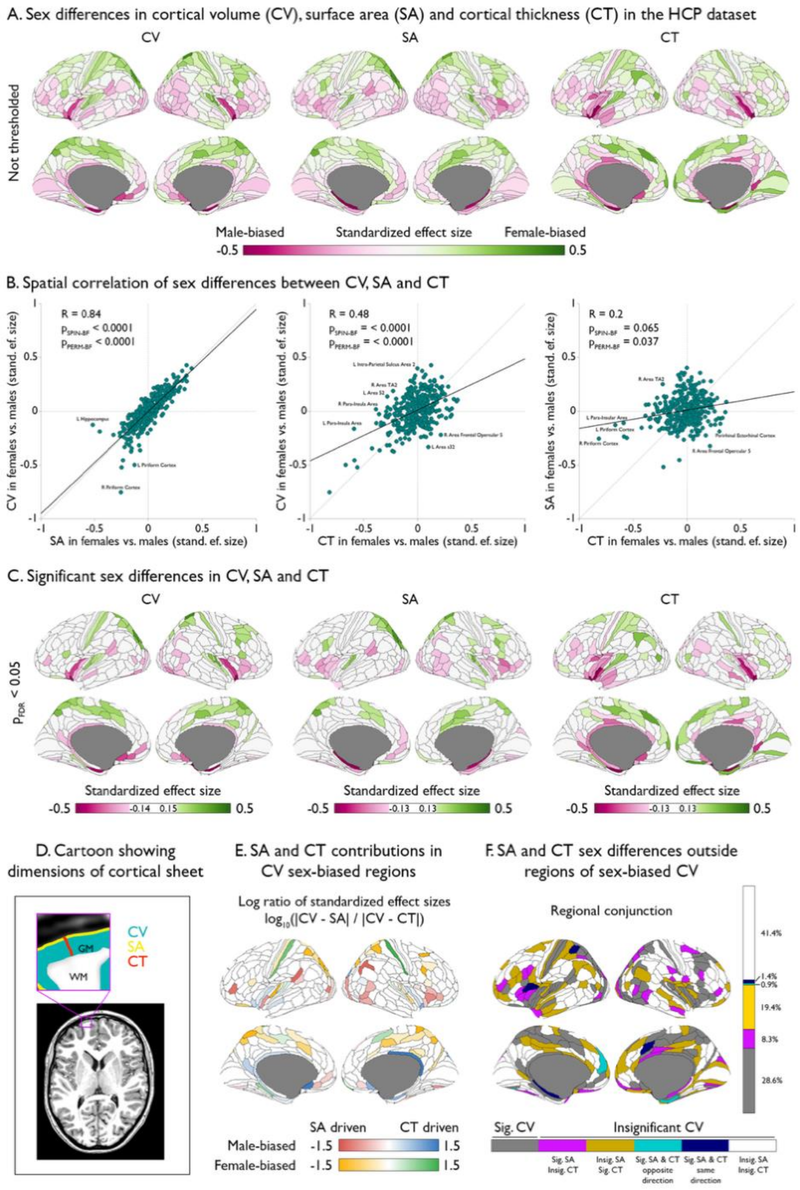

Figure 1 Caption, lines 149-150: "***D***. *Cartoon showing dimensions of cortical sheet, with CV, SA and CT highlighted in green, yellow and red (respectively).*"

Comment 6

If understanding Fig 1E correctly, it indicates that 58% of the regions (is that 58% of the 360 regions or 58% of the cortical sheet?) exhibit no significant sex differences in CV, SA or CT. If that is not the case please make clear what is, and if true, some discussion of those regions showing no sex differences would be of value to the reader.

Response 6

We thank the reviewer for this question – which has prompted us to revise the figure and text descriptions as reproduced below. This revised presentation now more explicitly conveys that 41% of the cortical sheet exhibits no significant sex differences in CV, SA or CT.

Results, lines 187-189: *"Specifically, 30% of the cortical sheet lacked a statistically significant sex difference in CV but showed a statistically significant sex difference in SA, CT or both these features (**Figure 1F**).*"

Results, lines 189-191: *"Conversely, 41% of cortical regions did not show a statistically significant sex difference in any of the 3 anatomical features considered. These regions included both unimodal (primary motor and visual cortices) and transmodal (components of the default mode network) cortices and overlap with regions that showed no sex difference in an independent meta-analysis*[39]."

Comment 7

Line 451 – (59% of cortical regions sex-biased considering all 3 features vs. 29% considering CV alone) – is this consistent with Figure 1 or is Figure 1 being misinterpreted by this reviewer?

Response 7

We appreciate the reviewer for raising this question. While our initial statement in line 451 is correct, the denominator for the percentages shown in Figure 1E was the cortical regions that show no significant sex differences in CV. In the revised Figure 1, the percentages are now based on the entire cortical sheet (= 360 cortical regions) as the denominator. It can now be readily interpreted from this revised figure that 29% of cortical regions sex-biased when considering CV alone, but 59% of cortical regions are sex-biased when considering all 3 features. Furthermore, subtracting these two percentages yields 30%, which is consistent with the statement in Response 6 that 30% of the cortical regions lacking significant sex difference in CV shows significant sex difference in SA, CT or both.

Please see the revised figure in our Response 5.

Comment 8

Discussion of the Results of Figure 1 notes bilateral sex differences in various regions, does this mean when the term “bilateral” is not used that there is a unilateral sex difference? Overall were there more bilateral than unilateral differences and was there any skewing by sex as to whether there is more of bilateral versus unilateral differences?

Response 8

Yes, the reviewer is right that regions mentioned without bilateral are unilateral. We agree the question of laterality is of great scientific interest, but space limitations currently prevent us from presenting the additional analyses that would be required to address the interesting questions raised by the reviewer.

Comment 9

For data in Fig 2, do the authors have any speculation why the variance in CT is notably smaller than that for SA and CV – is this a measurement artifact or a biological reality?

Response 9

We think this difference reflects the stronger constraints in interindividual variability of CT as compared to SA. Prior work has shown that the coefficient of variation across individuals is larger for SA (9.9%) than CT (3.2%)[40]. We have now cited this prior work in our Results section:

Results, lines 207-208: "*The variance in split-half correlations was notably lower for CT than CV and SA, which is consistent with the recognized stronger constraint on CT inter-individual variability*[40]."

Comment 10

The use of the Allen Brain Atlas for mapping transcriptional profiles onto regions with sex differences is compromised by the inclusion of only one female in the dataset. Just as we know that training AI on biased data results in biased AI, it would seem there is a high probability of the same happening here. This is especially true in light of this being post-mortem data and both the causes and course of death being highly influenced by gender. Studies by Nestler and others have found profound sex differences in postmortem human cortex in the context of major depressive disorder etc. More attention to this caveat and some strategy for avoiding the early introduction of bias to the analyses would be beneficial.

Response 10

We thank the reviewer for raising this important concern. We agree that the predominance of males in the Allen Human Brain Atlas is a source of bias in our analyses and replicating our transcriptomic annotations using a sex-balanced dataset will be needed to address this limitation. We have revised the discussion on the transcriptomic annotations in Discussions as below:

Discussions, lines 512-518: "*However, an important caveat to our transcriptional annotation of neuroanatomical sex difference maps is that the reference transcriptomic dataset*[41] *only contains one female and 5 males. As such, those significant transcriptomic correlations that we do recover should primarily be interpreted as regionally varying genes in males that track with neuroanatomical sex differences between males and females – which still offers useful insights. Definitively addressing this inferential limit awaits access to spatially comprehensive maps of regional cortical gene expression from large sex-balanced samples.*"

Comment 11

Along these same lines, what was the sex distribution of subjects used to create the Yeo-Krienen 17 resting-state functional networks? Could there be similar sources of bias here?

Response 11

The Yeo-Krienen 17 resting-state functional networks were based on the same HCP cohort used in our study.

Minor Comments:

Comment 12

Is the sex difference in SA a by-product of the difference in overall brain size?

Response 12

We included total surface area as a covariate in our analyses of regional SA sex differences. Total SA is the global phenotype through which brain size effects are conveyed to regional SA and is a stricter correction factor than total brain volume in analyses of regional SA. So, the regional SA sex differences we identity exist above and beyond variations in overall brain size.

Comment 13

What is “split-half sample size”

Response 13

It is the sample size after splitting the sample into two equal-sized halves. In our analyses, a split-half sample size of 400 means that there are two half-samples, each with 400 individuals (200 males and 200 females). As such, sex effect maps computed in one half-sample of 400 individuals are compared to those computed in the other half-sample of 400 individuals.

Comment 14

What was the mean age for those with sex chromosome aneuploidies and IGD compared to controls?

Response 14

Please see our **Supplementary Table 1**, which indicates the mean age of XXY, XYY and IGD case groups as well as their control groups.

Comment 15

Line 497 – “statistically surprising tendency”, should this be “surprising statistical trend”?

Response 15

We thank the reviewer for raising this point.

We have revised the phrase to “surprising statistical trend” as suggested as below:

Discussions, line 547: “…*surprising statistical trend* for regions of sex-biased anatomy…”

References

1 Bethlehem, R. A. I. *et al.* Brain charts for the human lifespan. *Nature* **604**, 525-533 (2022). https://doi.org/10.1038/s41586-022-04554-y

2 Potvin, O., Dieumegarde, L. & Duchesne, S. Normative morphometric data for cerebral cortical areas over the lifetime of the adult human brain. *NeuroImage* **156**, 315-339 (2017). https://doi.org/https://doi.org/10.1016/j.neuroimage.2017.05.019

3 Peper, J. S., Burke, S. M. & Wierenga, L. M. in *Handbook of Clinical Neurology* Vol. 175 (eds Rupert Lanzenberger, Georg S. Kranz, & Ivanka Savic) 25-54 (Elsevier, 2020).

4 Lenroot, R. K. & Giedd, J. N. Brain development in children and adolescents: insights from anatomical magnetic resonance imaging. *Neuroscience & biobehavioral reviews* **30**, 718-729 (2006).

5 Kaczkurkin, A. N., Raznahan, A. & Satterthwaite, T. D. Sex differences in the developing brain: insights from multimodal neuroimaging. *Neuropsychopharmacology* **44**, 71-85 (2019). https://doi.org/10.1038/s41386-018-0111-z

6 Giedd, J. N., Raznahan, A., Mills, K. L. & Lenroot, R. K. Review: magnetic resonance imaging of male/female differences in human adolescent brain anatomy. *Biology of Sex Differences* **3**, 19 (2012). https://doi.org/10.1186/2042-6410-3-19

7 Fish, A. M. *et al.* Sex-biased trajectories of amygdalo-hippocampal morphology change over human development. *NeuroImage* **204**, 116122 (2020). https://doi.org/https://doi.org/10.1016/j.neuroimage.2019.116122

8 De Bellis, M. D. *et al.* Sex Differences in Brain Maturation during Childhood and Adolescence. *Cerebral Cortex* **11**, 552-557 (2001). https://doi.org/10.1093/cercor/11.6.552

9 Kundakovic, M. & Tickerhoof, M. Epigenetic mechanisms underlying sex differences in the brain and behavior. *Trends in Neurosciences* **47**, 18-35 (2024). https://doi.org/10.1016/j.tins.2023.09.007

10 Tooley, U. A., Bassett, D. S. & Mackey, A. P. Environmental influences on the pace of brain development. *Nature Reviews Neuroscience* **22**, 372-384 (2021). https://doi.org/10.1038/s41583-021-00457-5

11 Portengen, C. M., van Baar, A. L. & Endendijk, J. J. A neurocognitive approach to studying processes underlying parents' gender socialization. *Frontiers in Psychology* **13**, 1054886 (2023).

12 Wu, H. *et al.* Occupational neuroplasticity in the human brain: a critical review and meta-analysis of neuroimaging studies. *Frontiers in human neuroscience* **14**, 215 (2020).

13 Forbes, C. E. & Leitner, J. B. Stereotype threat engenders neural attentional bias toward negative feedback to undermine performance. *Biological Psychology* **102**, 98-107 (2014). https://doi.org/https://doi.org/10.1016/j.biopsycho.2014.07.007

14 Zugman, A. *et al.* Country-level gender inequality is associated with structural differences in the brains of women and men. *Proceedings of the National Academy of Sciences* **120**, e2218782120 (2023). https://doi.org/doi:10.1073/pnas.2218782120

15 Raznahan, A. *et al.* Globally Divergent but Locally Convergent X- and Y-Chromosome Influences on Cortical Development. *Cerebral Cortex* **26**, 70-79 (2014). https://doi.org/10.1093/cercor/bhu174

16 Seidlitz, J. *et al.* Transcriptomic and cellular decoding of regional brain vulnerability to neurogenetic disorders. *Nature Communications* **11**, 3358 (2020). https://doi.org/10.1038/s41467-020-17051-5

17 Shanmugan, S. *et al.* Sex differences in the functional topography of association networks in youth. *Proceedings of the National Academy of Sciences* **119**, e2110416119 (2022). https://doi.org/doi:10.1073/pnas.2110416119
18 Segal, A. *et al.* Regional, circuit and network heterogeneity of brain abnormalities in psychiatric disorders. *Nature Neuroscience* **26**, 1613-1629 (2023). https://doi.org/10.1038/s41593-023-01404-6
19 Levitis, E. *et al.* The Variegation of Human Brain Vulnerability to Rare Genetic Disorders and Convergence With Behaviorally Defined Disorders. *Biological Psychiatry* **95**, 136-146 (2024). https://doi.org/10.1016/j.biopsych.2023.07.008
20 Goodkind, M. *et al.* Identification of a common neurobiological substrate for mental illness. *JAMA psychiatry* **72**, 305-315 (2015).
21 Manara, R. *et al.* Brain Changes in Kallmann Syndrome. *American Journal of Neuroradiology* **35**, 1700-1706 (2014). https://doi.org/10.3174/ajnr.A3946
22 Mallard, T. T. *et al.* X-chromosome influences on neuroanatomical variation in humans. *Nature Neuroscience* **24**, 1216-1224 (2021). https://doi.org/10.1038/s41593-021-00890-w
23 Cole, K. M. *et al.* The NIMH Intramural Longitudinal Study of the Endocrine and Neurobiological Events Accompanying Puberty: Protocol and rationale for methods and measures. *NeuroImage* **234**, 117970 (2021). https://doi.org/https://doi.org/10.1016/j.neuroimage.2021.117970
24 Heller, C. *et al.* Hormonal milieu influences whole-brain structural dynamics across the menstrual cycle using dense sampling in multiple individuals. *Nature neuroscience* **28**, 2588-2600 (2025).
25 Rizor, E. J. *et al.* Menstrual cycle-driven hormone concentrations co-fluctuate with white and gray matter architecture changes across the whole brain. *Human Brain Mapping* **45**, e26785 (2024).
26 Küchenhoff, S. *et al.* Relating sex-bias in human cortical and hippocampal microstructure to sex hormones. *Nature Communications* **15**, 7279 (2024). https://doi.org/10.1038/s41467-024-51459-7
27 Pritschet, L. *et al.* Neuroanatomical changes observed over the course of a human pregnancy. *Nature Neuroscience* **27**, 2253-2260 (2024). https://doi.org/10.1038/s41593-024-01741-0
28 Martínez-García, M., Jacobs, E. G., de Lange, A.-M. G. & Carmona, S. Advancing the neuroscience of human pregnancy. *Nature Neuroscience* **27**, 805-807 (2024). https://doi.org/10.1038/s41593-024-01629-z
29 Mosconi, L. *et al.* Menopause impacts human brain structure, connectivity, energy metabolism, and amyloid-beta deposition. *Scientific Reports* **11**, 10867 (2021). https://doi.org/10.1038/s41598-021-90084-y
30 Anawalt, B. D. & Merriam, G. R. NEUROENDOCRINE AGING IN MEN: Andropause and Somatopause. *Endocrinology and Metabolism Clinics of North America* **30**, 647-669 (2001). https://doi.org/https://doi.org/10.1016/S0889-8529(05)70206-1
31 Ogoniak, L. *et al.* Role of genetics in the age-related testosterone decline in men: a UK Biobank study. *European Journal of Endocrinology* **193**, 197-203 (2025). https://doi.org/10.1093/ejendo/lvaf143
32 Raznahan, A. & Disteche, C. M. X-chromosome regulation and sex differences in brain anatomy. *Neuroscience & Biobehavioral Reviews* **120**, 28-47 (2021). https://doi.org/https://doi.org/10.1016/j.neubiorev.2020.10.024

33 Raznahan, A. *et al.* Triangulating the sexually dimorphic brain through high-resolution neuroimaging of murine sex chromosome aneuploidies. *Brain Structure and Function* **220**, 3581-3593 (2015). https://doi.org/10.1007/s00429-014-0875-9
34 Warrier, V. *et al.* Genetic insights into human cortical organization and development through genome-wide analyses of 2,347 neuroimaging phenotypes. *Nat Genet* **55**, 1483-1493 (2023). https://doi.org/10.1038/s41588-023-01475-y
35 Grotzinger, A. D. *et al.* Multivariate genomic architecture of cortical thickness and surface area at multiple levels of analysis. *Nature Communications* **14**, 946 (2023). https://doi.org/10.1038/s41467-023-36605-x
36 Panizzon, M. S. *et al.* Distinct Genetic Influences on Cortical Surface Area and Cortical Thickness. *Cerebral Cortex* **19**, 2728-2735 (2009). https://doi.org/10.1093/cercor/bhp026
37 Rakic, P. Specification of cerebral cortical areas. *Science* **241**, 170-176 (1988).
38 Rash, B. G. & Grove, E. A. Area and layer patterning in the developing cerebral cortex. *Current Opinion in Neurobiology* **16**, 25-34 (2006). https://doi.org/https://doi.org/10.1016/j.conb.2006.01.004
39 Ruigrok, A. N. V. *et al.* A meta-analysis of sex differences in human brain structure. *Neuroscience & Biobehavioral Reviews* **39**, 34-50 (2014). https://doi.org/https://doi.org/10.1016/j.neubiorev.2013.12.004
40 Winkler, A. M. *et al.* Joint Analysis of Cortical Area and Thickness as a Replacement for the Analysis of the Volume of the Cerebral Cortex. *Cerebral Cortex* **28**, 738-749 (2017). https://doi.org/10.1093/cercor/bhx308
41 Hawrylycz, M. J. *et al.* An anatomically comprehensive atlas of the adult human brain transcriptome. *Nature* **489**, 391-399 (2012).

Reviewer 1's Comment

Authors have addressed all of my concerns.

Response

We thank Dr. Elvisha Dhamala for their constructive comments and suggestions that allowed us to substantially improve the results, interpretations, and discussions of the manuscript.

Reviewer 2's Comment

The authors have done an excellent job addressing prior noted concerns. The revisions are clear, thorough, and responsive to the original feedback. I am satisfied with the current version of the manuscript and have no further comments. I recommend this manuscript for publication.

Response

We are delighted and grateful for the reviewer's comment. The reviewer's constructive comments have greatly contributed to improving the presentation, interpretation, and discussion of the study.

Reviewer 3's Comment

The authors have addressed all my concerns in a satisfactorily manner.

Response

We thank the reviewer for raising constructive points, which led us to improve the interpretation of our findings and highlight some of the important steps needed to address the gaps in this line of research.

Reviewer 4's Comment

The authors have done an excellent job responding to both mine and other reviewers' suggestions for improvement, being both gracious and responsive. I did note there was no response to the request from Rev 3 (?) to discuss the absence of XO females and would be curious to hear the authors views on whether these individuals would be informative as to potential

feminizing effects of estrogen as suggested by the reviewer, but this is in no way necessary for the publication which in my view can be accepted as is.

Response

We thank the reviewer for their constructive feedback that allowed us to greatly increase the accessibility of the manuscript, including its title.

In the previous round of revision, our response to Reviewer 2's Comment 3 was also meant to serve as a response to the Reviewer 3's comment for the absence of XO females, but we did not indicate this intention for Reviewer 3.

We believe that the X-dosage effects on regional brain anatomy observed in the contrast between XXY and XY individuals largely reflect direct and gonad independent influences impact of X chromosome rather than the endocrine abnormalities in the context sex chromosome aneuploidies. This view is informed by the prior published evidence for consistent neuroanatomical effects of X-dosage across multiple sex chromosome aneuploidies (XXX, XXXX, XXYY, XXXXY, and XO) irrespective of the gonadal status. However, we very much agree that the potential feminizing effects of estrogen and broader ovarian function are of great interest.
